# Recent Updates in Experimental Research and Clinical Evaluation on Drugs for COVID-19 Treatment

**DOI:** 10.3389/fphar.2021.732403

**Published:** 2021-11-22

**Authors:** Houwen Zou, Yuqi Yang, Huiqiang Dai, Yunchuang Xiong, Jing-Quan Wang, Lusheng Lin, Zhe-Sheng Chen

**Affiliations:** ^1^ Ganzhou Cancer Hospital, Ganzhou, China; ^2^ Department of Pharmaceutical Sciences, College of Pharmacy and Health Sciences, St. John’s University, Queens, NY, United States; ^3^ Cell Research Center, Shenzhen Bolun Institute of Biotechnology, Shenzhen, China

**Keywords:** corona virus disease 2019, corona virus disease 2019 treatment, severe acute respiratory syndrome corona virus 2 variants, antimicrobials, immunotherapy, traditional Chinese medicine

## Abstract

Since the outbreak of corona virus disease 2019 (COVID-19) in Wuhan (China) in December 2019, the epidemic has rapidly spread to many countries around the world, posing a huge threat to global public health. In response to the pandemic, a number of clinical studies have been initiated to evaluate the effect of various treatments against COVID-19, combining medical strategies and clinical trial data from around the globe. Herein, we summarize the clinical evaluation about the drugs mentioned in this review for COVID-19 treatment. This review discusses the recent data regarding the efficacy of various treatments in COVID-19 patients, to control and prevent the outbreak.

## Introduction

The outbreak of corona virus disease 2019 (COVID-19), from Wuhan, Hubei Province, China, in December 2019, has now become the first global pandemic caused by the spread of coronavirus. On February 11, 2020, the World Health Organization (WHO) gave a name for the novel coronavirus as severe acute respiratory syndrome coronavirus 2 (SARS-CoV-2) and the coronavirus disease of 2019 (COVID-19) caused by SARS-CoV-2 (Bevova et al., 2020). Most recently, several predominate SARS-CoV-2 variants, including, but not limited to, B.1.1.7 (alpha) variant, B.1.351 (beta) variant, P.1 (gamma), and B.1.617.2 (delta) variant, were first detected in the United Kingdom, South Africa, Brazil, and India, and became a novel global concern (Ong et al., 2021; Sanches et al., 2021). The SARS-CoV-2 variants have greater ability of virus infectivity and immune escape, suggesting that the SARS-CoV-2 variants may result in poor treatment efficacy and prognosis for COVID-19 patients. In the past few months, many research teams from around the world have been conducting *in vitro* and *in vivo* studies of the virus, seeking effective prevention and control measures to prevent its spread. China is relatively fast and effective in the control of epidemic. We are, therefore, able to comprehensively analyze common domestic treatment methods and combined domestic and foreign research to jointly explore effective treatment programs for COVID-19, to provide guidance for the second wave of the epidemic.

Many products including, but not limited to, traditional Chinese medicines (TCMs), antiviral drugs (e.g., chloroquine phosphate and alpha-interferon) (Wang and Zhu, 2020), monoclonal antibodies (e.g., tozumab combined with adamumab) (Sarkar et al., 2020), convalescence plasma, and mesenchymal stem cells (MSCs) (Peng et al., 2020) have become the focus of our communication for COVID-19 treatment. The Chinese Clinical Trial Registry (ChiCTR) shows a large number of TCM-related drugs studied for the treatment and prevention of COVID-19 (e.g., Lianhuaqingwen capsule, Shuanghuanglian oral liquid, Xuebijing injection, etc.) (Li H. et al., 2020), while Western drugs focus on antiviral drugs and immunotherapy (e.g., stem cell-based therapy, antibodies, etc.) (Ni et al., 2020a; Gulati et al., 2021). In the Diagnosis and Treatment Protocol for Novel Coronavirus Pneumonia (Trial Version 7), it mentioned several antiviral drugs such as chloroquine (CQ), alpha-interferon (IFN-α), lopinavir/ritonavir, and umifenovir, and also mentioned immunotherapy, such as tocilizumab (Wei, 2020a; Zhao et al., 2020a; Wei, 2020b). Notably, as the adage, “old drug, new trick,” most of the antiviral drugs used for COVID-19 treatment are existing compounds screened for potential use based on mechanistic similarities to other viruses.

**FIGURE 1:**
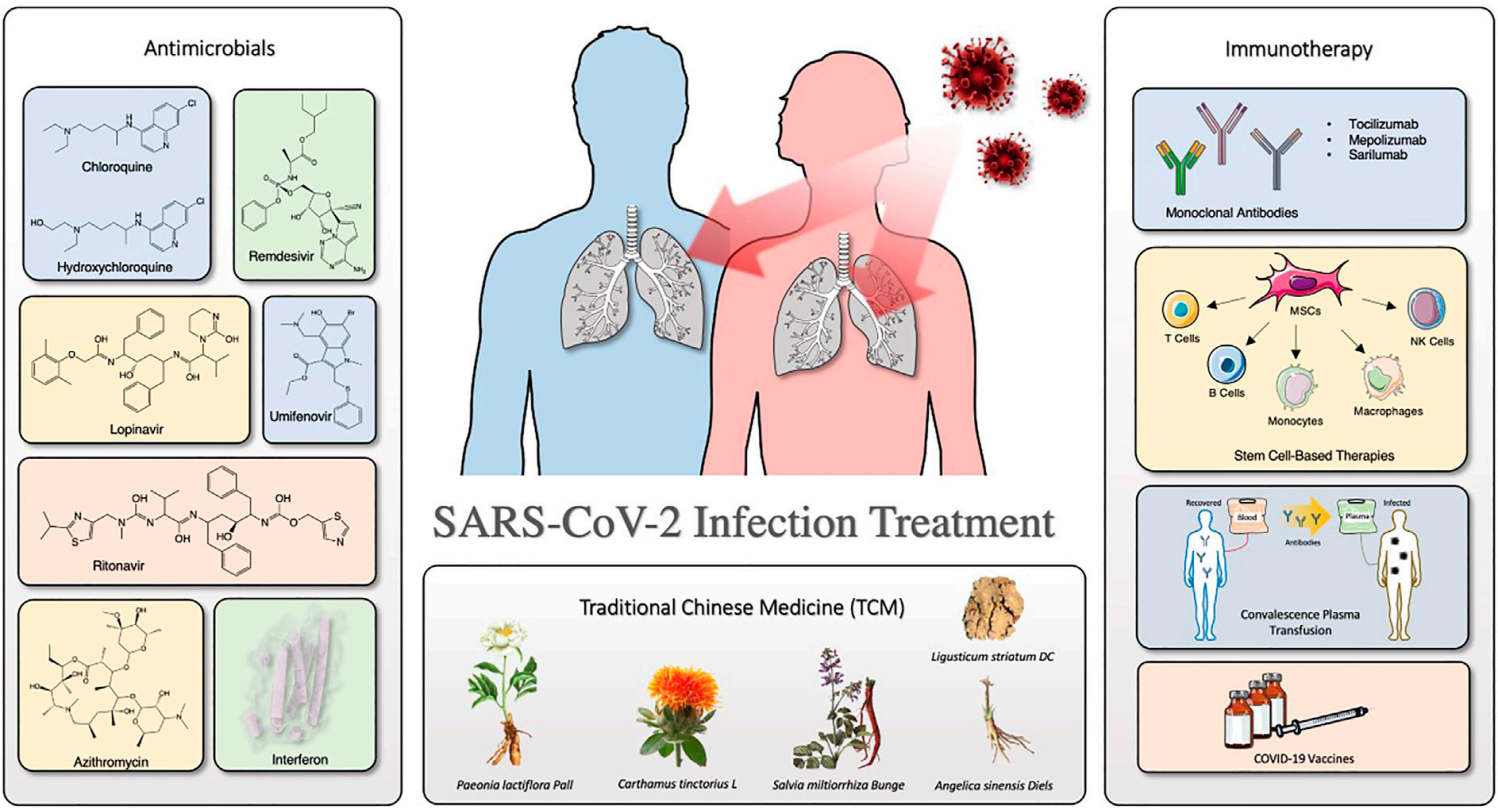
Overview of the organization of this review.

Herein, we summarize the clinical evaluation for COVID-19 treatment about the drugs mentioned in this review. Figure 1 depicts the overview of the organization of this review. Furthermore, we discuss recent representative progresses and considerations in the treatment for COVID-19, especially antimicrobials (antivirals and antibiotics/antibacterial), immunotherapy, and TCMs.

## Antimicrobials

### Chloroquine and hydroxychloroquine

As a widely used antimalarial and immunomodulatory drug, chloroquine (CQ) shows a broad-spectrum antiviral activity. Table 1 summarizes the clinical trials of CQ and HCQ for the treatment of COVID-19. Some researchers indicated that CQ is effective against SARS-CoV-2 virus in early clinical studies (Huang et al., 2020c). Of note, chloroquine phosphate is undergoing some clinical trials regarding prophylactic use in health personnel (Clinicaltrials.gov, NCT04443270) and against infection by SARS-CoV-2 (*Clinicaltrials.gov*, NCT04344951). Evidence from a multicenter prospective observational study indicated that patients in CQ treatment group have shorter median time to achieve an undetectable viral RNA and shorter duration of fever; also, more importantly, no severe side effects were found during CQ treatment (Huang et al., 2020b). Hydroxychloroquine (HCQ) is an analog of CQ by replacing an ethyl group in CQ with a hydroxyethyl group (Zhou et al., 1878). Nowadays, ChiCTR conducts many clinical trials in China to examine the effectiveness and safety of CQ or HCQ against COVID-19 (Gao et al., 2020). A team from Renmin Hospital of Wuhan University investigated the effects of HCQ among 62 patients suffering from COVID-19 (www.chictr.org.cn, ChiCTR2000029559). As a result, Chen et al. found that HCQ could significantly shorten time to clinical recovery (TTCR) and improve pneumonia.

**TABLE 1:** Summary of clinical trials of chloroquine (CQ) and hydroxychloroquine (HCQ) on COVID-19 treatment.

Trial title	Sponsor	Trial phase	Primary intervention	Secondary intervention	Status	Identifier
Efficacy of Chloroquine or Hydroxychloroquine in COVID-19 Treatment	Tanta University	II/III	Chloroquine or hydroxychloroquine	N/A	Recruiting	NCT04353336
Chloroquine for Mild Symptomatic and Asymptomatic COVID-19	HaEmek Medical Center, Israel	II/III	Chloroquine	Standard care	Terminated (Terminated due to changes in treatment guidelines.)	NCT04333628
Zinc With Chloroquine/Hydroxychloroquine in Treatment of COVID-19	Tanta University	III	Chloroquine	Chloroquine + zinc tablets	Recruiting	NCT04447534
The Vietnam Chloroquine Treatment on COVID-19 (VICO)	Oxford University Clinical Research Unit, Vietnam	II	Chloroquine phosphate	N/A	Recruiting	NCT04328493
Chloroquine + Losartan Compared to Chloroquine Alone for the Treatment of COVID-19 Pneumonia	Hospital Universitario Dr. Jose E. Gonzalez	II	Chloroquine phosphate	Chloroquine + losartan	Withdrawn (Evidence showed chloroquine is not effective against COVID-19.)	NCT04428268
Chloroquine Phosphate Prophylactic Use in Health Personnel Exposed to COVID-19 Patients	CMN ″20 de Noviembre"	I	Chloroquine phosphate	N/A	Not yet recruiting	NCT04443270
Prevention with Chloroquine in Health Personnel Exposed to Infection with Coronavirus Disease 2019 (COVID-19) (TS-COVID) (TS-COVID)	Fundacion Clinica Valle del Lili	II	Chloroquine	N/A	Active, not recruiting	NCT04627467
Chloroquine Phosphate Against Infection by the Novel Coronavirus SARS-CoV-2 (COVID-19): The HOPE Open-Label, Non-Randomized Clinical Trial (HOPE)	Uni-Pharma Kleon Tsetis Pharmaceutical Laboratories S.A.	II	Chloroquine	N/A	Recruiting	NCT04344951
Chloroquine Outpatient Treatment Evaluation for HIV-COVID-19 (CQOTE)	University of Cape Town	III	Chloroquine or hydroxychloroquine	Standard care	Withdrawn (Equipoise for hydroxychloroquine was lost.)	NCT04360759
Chloroquine as Antiviral Treatment in Coronavirus Infection 2020	Wroclaw Medical University	IV	Chloroquine phosphate	Telemedicine	Completed	NCT04331600
Chloroquine, Hydroxychloroquine or Only Supportive Care in Patients Admitted With Moderate to Severe COVID-19 (ARCHAIC)	UMC Utrecht	IV	Chloroquine sulfate or hydroxychloroquine	Supportive care only	Terminated (Currently, almost no patients are admitted to Dutch hospitals. If any effect of HCQ is to be expected, we need more than 1,000 inclusions.)	NCT04362332
Chloroquine Diphosphate in the Prevention of SARS in COVID-19 Infection (CloroCOVID19II)	Fundação de Medicina Tropical Dr. Heitor Vieira Dourado	II	Chloroquine diphosphate	Placebo oral tablet	Completed	NCT04342650
Efficacy of Chloroquine or Hydroxychloroquine in Treating Pneumonia Caused by SARS-Cov-2-COVID-19	Centro de Estudos e Pesquisa em Emergencias medicase Terapia Intensiva	III	Chloroquine or hydroxychloroquine	Standard care	Completed	NCT04420247
Saved From COVID-19	Columbia University	II	Chloroquine	Placebo oral tablet	Terminated (Low enrollment.)	NCT04349371
Prophylaxis of Exposed COVID-19 Individuals With Mild Symptoms Using Chloroquine Compounds (PRECISE)	Government of Punjab, Specialized Healthcare and Medical Education Department	IV	Chloroquine or hydroxychloroquine	Standard of care + placebo	Terminated (Poor accrual.)	NCT04351191
The Clinical Study of Carrimycin on Treatment Patients With COVID-19	Beijing YouAn Hospital	IV	Carrimycin	Chloroquine phosphate or lopinavir/ritonavir tablets or arbidol	Not yet recruiting	NCT04286503
Post-Exposure Prophylaxis for Asymptomatic SARS-CoV-2 COVID-19 Patients With Chloroquine Compounds (PEACE)	Government of Punjab, Specialized Healthcare and Medical Education Department	IV	Chloroquine or hydroxychloroquine	Standard of care + placebo	Terminated (Poor accrual.)	NCT04346667
Immune Monitoring of Prophylactic Effect of Hydroxychloroquine in Healthcare Providers Highly Exposed to COVID-19 (Chloroquine UN)	Universidad Nacional de Colombia	III	Hydroxychloroquine	Placebo oral tablet	Withdrawn (The study did not get financed. Never got started.)	NCT04346329
Angiotensin Converting Enzyme Inhibitors in Treatment of Covid 19	Tanta University	III	Captopril or enalapril	Chloroquine	Not yet recruiting	NCT04345406
Evaluation of Efficacy of Levamisole and Formoterol + Budesonide in Treatment of COVID-19	Fasa University of Medical Sciences	II/III	Levamisole pill + budesonide + formoterol inhaler	Hydroxychloroquine + lopinavir/ritonavir	Recruiting	NCT04331470
Austrian CoronaVirus Adaptive Clinical Trial (COVID-19) (ACOVACT)	Medical University of Vienna	II/III	Chloroquine or hydroxychloroquine	Lopinavir/ritonavir; Standard care	Recruiting	NCT04351724
An Adaptive Study of Favipiravir Compared to Standard of Care in Hospitalized Patients With COVID-19	Chromis LLC	II/III	Favipiravir	Chloroquine, hydroxychloroquine, lopinavir/ritonavir, etc.	Active, not recruiting	NCT04434248
Efficacy of Natural Honey Treatment in Patients With Novel Coronavirus	Misr University for Science and Technology	III	Natural honey	Chloroquine phosphate or hydroxychloroquine or lopinavir/ritonavir tablets or arbidol or oseltamivir with or without azithromycin	Recruiting	NCT04323345
Study of Favipiravir Compared to Standard of Care in Hospitalized Patients With COVID-19	Promomed, LLC	III	Favipiravir	Chloroquine, hydroxychloroquine, lopinavir/ritonaviretc.	Completed	NCT04542694
Hydroxychloroquine for Treatment of Non-Severe COVID-19 (HONEST)	Makerere University	II	Hydroxychloroquine tablets	Standard care only	Active, not recruiting	NCT04860284
Efficacy and Safety of Anti HCV Drugs in the Treatment of COVID-19	Cairo University	II/III	Hydroxychloroquine + sofosbuvir/daclatasvir	Hydroxychloroquine	Not yet recruiting	NCT04443725
Hydroxychloroquine as Post Exposure Prophylaxis for SARS-CoV-2(HOPE Trial)	Gangnam Severance Hospital	III	Hydroxychloroquine	N/A	Not yet recruiting	NCT04330144
PATCH 2 and 3: Prevention and Treatment of COVID-19 (Severe Acute Respiratory Syndrome Coronavirus 2) With Hydroxychloroquine	United Health Group	II	Hydroxychloroquine	Placebo drug	Terminated (As enrollment began, external studies called into question the safety and efficacy of hydroxychloroquine as a treatment, which resulted in controversy. The timing of the controversy significantly impacted our ability to enroll and retain participants.)	NCT04353037
Efficacy of Azithromycin-Associated Hydroxychloroquine Therapy Given in General Practice in Early-Stage Disease in COVID-19 Patients (MG-COVID)	Assistance Publique—Hôpitaux de Paris	III	Hydroxychloroquine + azithromycin	Dietary supplement: Azinc	Withdrawn (Regulatory approvals have not been obtained.)	NCT04371406
Evaluation of the Efficacy of the Hydroxychloroquine-Azithromycin Combination in the Prevention of COVID-19 Related Sdra (Teachcovid)	University Hospital, Strasbourg, France	III	Hydroxychloroquine and azithromycin	Hydroxychloroquine; IV antibiotics and standard of care	Withdrawn (In view of the notices concerning hydroxychloroquine issued by the regulatory authorities, we withdraw the protocol.)	NCT04347512
Safety and Efficacy of Hydroxychloroquine Associated With Azithromycin in SARS-CoV2 Virus (Coalition COVID-19 Brasil II)	Hospital Israelita Albert Einstein	III	Hydroxychloroquine + azithromycin	Hydroxychloroquine	Completed	NCT04321278
Hydroxychloroquine for the Treatment of Mild COVID-19 Disease (COMIHY)	University Hospital Tuebingen	II	Hydroxychloroquine	Placebo capsules	Terminated (It appeared to be impossible for the study lefts to recruit the targeted number of patients, due to reduced incidence and reduced acceptance to IMP.)	NCT04340544
Hydroxychloroquine for COVID-19 (COV-HCQ)	University Hospital Tuebingen	III	Hydroxychloroquine sulfate	Placebo capsules	Terminated (Reduced acceptance of IMP.)	NCT04342221
Hydroxychloroquine Versus Placebo in COVID-19 Patients at Risk for Severe Disease (HYCOVID)	University Hospital, Angers	III	Hydroxychloroquine	Placebo	Terminated (Decrease in number of eligible patients.)	NCT04325893
Hydroxychloroquine and Ivermectin for the Treatment of COVID-19 Infection	Centenario Hospital Miguel Hidalgo	III	Hydroxychloroquine	Ivermectin; placebo	Completed	NCT04391127
Assessing Hydroxychloroquine in Patients With SARS-CoV-2 (COVID-19)	Oregon Health and Science University	II	Hydroxychloroquine	Placebo	Withdrawn (Discontinued in favor of more promising directions that may benefit patients.)	NCT04363866
Healthcare Worker Exposure Response and Outcomes of Hydroxychloroquine (HERO-HCQ)	Adrian Hernandez	III	Hydroxychloroquine	Placebo oral tablet	Completed	NCT04334148
Favipiravir in Hospitalized COVID-19 Patients (FIC)	Shahid Beheshti University of Medical Sciences	IV	Favipiravir	Hydroxychloroquine	Not yet recruiting	NCT04359615
Hydroxychloroquine and lopinavir/ritonavir to Improve the Health of People With COVID-19: "The Hope Coalition - 1″	Cardresearch	III	Hydroxychloroquine sulfate tablets	Lopinavir/ritonavir oral tablet; placebo	Recruiting	NCT04403100
Chemoprophylaxis of SARS-CoV-2 Infection (COVID-19) in Exposed Healthcare Workers (COVIDAXIS)	Centre Hospitalier Universitaire de Saint Etienne	III	Hydroxychloroquine oral tablet	Lopinavir/ritonavir oral tablet; placebo	Active, not recruiting	NCT04328285
Azithromycin in Hospitalized COVID-19 Patients (AIC)	Shahid Beheshti University of Medical Sciences	IV	Hydroxychloroquine	Azithromycin	Not yet recruiting	NCT04359316

(All information in the table are collected from https://clinicaltrials.gov).

However, the high-quality clinical data showing a clear and reliable benefit of CQ or HCQ remains limited. Also, the CQ or HCQ treatment could induce severe cardiac side effects, imped innate and adaptive antiviral immune responses, and cause some uncertain effects (Meyerowitz et al., 2020). Commonly, QT prolongation and torsade de Pointes (TdP) occur on patients who are administered with CQ or HCQ (Blignaut et al., 2019). Hence, before CQ and HCQ treatment, an initial cardiac evaluation is necessary for COVID-19 patients (Zhou W. et al., 2020). Also, several follow-up evaluations, such as regular ophthalmological examination and cardiac monitor, are suggested for patients with short- or long-term treatment (Knox and Owens, 1966). Thus, using CQ or HCQ as a COVID-19 treatment was controversial, which results from their ocular, cardiac, and neuro toxicities (Oren et al., 2020; Zou et al., 2020). Additionally, the certainty of evidence is low. Together, we would like to recommend monitoring the accumulative effect of long-term and/or high-dose CQ or HCQ in clinical settings. Also, researchers are not supposed to overstate or understate the clinical efficacy of CQ and HCQ on COVID-19 treatment.

### Lopinavir/ritonavir

Lopinavir/ritonavir tablets (brand name: Kaletra) are two structurally related protease inhibitors and works as antiretroviral agents (Cvetkovic and Goa, 2003). Table 2 summarizes the clinical trials of lopinavir/ritonavir on COVID-19 treatment. The mechanism of action of protease inhibitors is block cleavage in Gag and Gag-Pol, and result in producing immature and noninfectious virus particles (Adamson, 2012). Similar to CQ, lopinavir/ritonavir could act as potential antiviral agents against SARS *in vitro* and in patients with SARS infection (Chu et al., 2004). Also, lopinavir/ritonavir has favorable clinical outcome with the Middle East respiratory syndrome coronavirus (MERS-CoV) after MERS reported in 2012 (Mo and Fisher, 2016; Arabi et al., 2018). Evidence from randomized trials indicated that lopinavir/ritonavir might improve outcomes in severe and critical COVID-19 patients, but it may induce mortality (Verdugo-Paiva et al., 2020). Moreover, it is reported that lopinavir/ritonavir could only improve a minority of throat-swab nucleic acid results in hospitals (Liu et al., 2020). Also, Cao et al. revealed that no beneficial response or clinical improvement was observed after treatment with lopinavir/ritonavir in a randomized, controlled, open-label trial with 199 in hospital patients suffering from severe SARS-CoV-2 infection, even though improvement was found for some secondary endpoints (Cao et al., 2020; Stower, 2020). Together, the response of COVID-19 patients with lopinavir/ritonavir is not ideal and unfavorable. As the previous study showed, CQ had more potent effects to patients with COVID-19 than the use of lopinavir/ritonavir; hence, an ongoing clinical trial in China would like to access the effectiveness and safety of CQ and lopinavir/ritonavir for patients suffering from mild or general SARS-CoV-2 infection (www.chictr.org.cn, ChiCTR2000029741). Overall, available data on the anti-SARS-CoV-2 activity of lopinavir/ritonavir are still limited and investigational, thereby the clinical application of lopinavir/ritonavir should be considered and monitored carefully.

**TABLE 2:** Summary of clinical trials of lopinavir/ritonavir on COVID-19 treatment.

Trial title	Sponsor	Trial phase	Primary intervention	Secondary intervention	Status	Identifier
Comparison of Lopinavir/Ritonavir or Hydroxychloroquine in Patients With Mild Coronavirus Disease (COVID-19)	Asan Medical Center	II	Lopinavir/ritonavir tablet	Hydroxychloroquine sulfate tablet	Terminated (Terminated early because no patients were further enrolled since mid-Apr 2020.)	NCT04307693
COVID-19 Ring-based Prevention Trial With Lopinavir/Ritonavir (CORIPREV-LR)	Darrell Tan	III	Lopinavir/ritonavir	N/A	Recruiting	NCT04321174
Lopinavir/Ritonavir for the Treatment of COVID-19 Positive Patients With Cancer and Immune Suppression in the Last Year	OHSU Knight Cancer Institute	II	Lopinavir/ritonavir	Placebo	Withdrawn (Limited resources.)	NCT04455958
FLARE: Favipiravir ± Lopinavir: A RCT of Early Antivirals (FLARE)	University College, London	II	Favipiravir	Lopinavir/ritonavir; placebo	Recruiting	NCT04499677
Trial of Early Therapies During Non-Hospitalized Outpatient Window for COVID-19 (TREATNOW)	Vanderbilt University Medical Center	II	Lopinavir/ritonavir tablets	Placebo	Recruiting	NCT04372628
Combination Therapies to Reduce Carriage of SARS-Cov-2 and Improve Outcome of COVID-19 in Ivory Coast: A phase Randomized IIb Trial (INTENSE-COV)	ANRS, Emerging Infectious Diseases	II/III	Lopinavir/ritonavir	Lopinavir/ritonavir + telmisartan; lopinavir/ritonavir + atorvastatin	Recruiting	NCT04466241
Comparative Therapeutic Efficacy and Safety of Different Antiviral and Anti-Inflammatory Drugs in COVID-19 Patients	October 6 University	IV	Remdesivir + tocilizumab + lopinavir/ritonavir	Hydroxychloroquine + tocilizumab + ivermectin	Recruiting	NCT04779047
Evaluation of Additional Treatments for COVID-19: A Randomized Trial in Niger (TRASCOV)	Epicentre	III	Standard care + lopinavir/ritonavir	Standard care	Withdrawn (Epidemic dynamics.)	NCT04409483
Baricitinib Therapy in COVID-19	Fabrizio Cantini	II/III	Lopinavir/ritonavir tablets	N/A	Completed	NCT04358614
Efficacy of Pragmatic Same-Day COVID-19 Ring Prophylaxis for Adult Individuals Exposed to SARS-CoV-2 in Switzerland (COPEP)	Calmy Alexandra	III	Lopinavir/ritonavir	N/A	Completed	NCT04364022
Baricitinib in Symptomatic Patients Infected by COVID-19: An Open-Label, Pilot Study (BARI-COVID)	Hospital of Prato	II/III	Lopinavir/ritonavir	Antiviral and/or hydroxychloroquine	Not yet recruiting	NCT04320277
Preventing Pulmonary Complications in Surgical Patients at Risk of COVID-19 (PROTECT-Surg)	University of Birmingham	III	Lopinavir/ritonavir	Hydroxychloroquine; lopinavir/ritonavir + hydroxychloroquine	Not yet recruiting	NCT04386070
Trial of Treatments for COVID-19 in Hospitalized Adults (DisCoVeRy)	Institut National de la Santé Et de la Recherche Médicale, France	III	Lopinavir/ritonavir	Lopinavir/ritonavir + interferon β-1a; remdesivir; hydroxychloroquine; standard care; placebo	Active, not recruiting	NCT04315948
Treatment for COVID-19 in High-Risk Adult Outpatients	University of Washington	II/III	Lopinavir/ritonavir	Hydroxychloroquine + folic acid; hydroxychloroquine + azithromycin	Active, not recruiting	NCT04354428
Randomized Evaluation of COVID-19 Therapy (RECOVERY)	University of Oxford	II/III	Lopinavir/ritonavir	Corticosteroid; hydroxychloroquine; azithromycin; convalescent plasma; tocilizumab; immunoglobulin; synthetic neutralizing antibodies; aspirin; colchicine; baricitinib; anakinra; dimethyl fumarate; infliximab	Recruiting	NCT04366089
A Study of Combination Therapies to Treat COVID-19 Infection	ProgenaBiome	II	Hydroxychloroquine + lopinavir/ritonavir, + azithromycin	Hydroxychloroquine + azithromycin	Withdrawn (Was never started.)	NCT04459702
Isotretinoin in Treatment of COVID-19 (Randomized)	Tanta University	III	Isotretinoin	Paracetamol, hydroxychloroquine, oseltamivir, azithromycin or clarithromycin ascorbic acid and cyanocobalamin plus lopinavir/ritonavir with or without isotretinoin	Not yet recruiting	NCT04361422
P2Et Extract in the Symptomatic Treatment of Subjects With COVID-19	Hospital Universitario San Ignacio	II/III	Lopinavir/ritonavir, hydroxychloroquine, *Caesalpinia spinosa* extract capsule	Placebo	Recruiting	NCT04410510

(All information in the table are collected from https://clinicaltrials.gov).

### Remdesivir

Remdesivir (GS-5734, brand name: Veklury), as a nucleotide analog prodrug, is a broad-spectrum antiviral drug that acts on RNA-dependent RNA polymerase (RdRp) and results in premature termination (Tchesnokov et al., 2019; Lamb, 2020). Table 3 shows the summary of clinical trials of remdesivir on COVID-19 treatment. As previously mentioned, Wang et al. showed that the EC_50_ value of remdesivir is 1.76 μM in Vero E6 cells, which suggests that remdesivir has high effectiveness in the control of SARS-CoV-2 infection *in vitro* (Wang M. et al., 2020). More importantly, the intravenous remdesivir was administrated to the patient who was the first case diagnosed as SARS-CoV-2 infection in the United States (Holshue et al., 2020). No adverse effects were observed in association with the infusion; also, clinical benefits were found in patients. Another case demonstrated that remdesivir could accelerate recovery time by 4 days, which is a meaningful and optimistic progress for patients and medical systems (Jorgensen et al., 2020). Notably, remdesivir is FDA approved specifically for the treatment of COVID-19. However, as more and more clinical cases were reported, the outcome of remdesivir treatment sometimes cannot achieve the expected effects on COVID-19 patients. Many researchers (Wang Y. et al., 2020) carried out a randomized, double-blind, placebo-controlled, multicenter trial; as a result, Wang et al. found that remdesivir is not associated with statistically significant clinical improvement, even though some patients in the remdesivir treatment group had numerically faster time to improve than those in the placebo group. More importantly, remdesivir treatment was discontinued early due to the adverse events, including, but not limited to, nausea, constipation, and respiratory failure or acute respiratory distress. Overall, the certainty of evidence remains less. Since Nov. 2020, the WHO has issued a conditional recommendation against the use of remdesivir in COVID-19 patients.

**TABLE 3:** Summary of clinical trials of remdesivir on COVID-19 treatment.

Trial title	Sponsor	Trial phase	Primary intervention	Secondary intervention	Status	Identifier
Study to Evaluate the Safety, Tolerability, Pharmacokinetics, and Efficacy of Remdesivir (GS-5734™) in Participants From Birth to <18 Years of Age With Coronavirus Disease 2019 (COVID-19) (CARAVAN)	Gilead Sciences	II/III	Remdesivir	N/A	Recruiting	NCT04431453
Remdesivir Efficacy in Coronavirus Disease	Tanta University	II/III	Remdesivir	Standard of care	Recruiting	NCT04345419
Study in Participants With Early-Stage Coronavirus Disease 2019 (COVID-19) to Evaluate the Safety, Efficacy, and Pharmacokinetics of Remdesivir Administered by Inhalation	Gilead Sciences	I/II	Remdesivir	Placebo	Completed	NCT04539262
A Trial of Remdesivir in Adults With Severe COVID-19	Capital Medical University	III	Remdesivir	Placebo	Terminated (The epidemic of COVID-19 has been controlled well in China; no eligible patients can be enrolled at present.)	NCT04257656
A Trial of Remdesivir in Adults With Mild and Moderate COVID-19	Capital Medical University	III	Remdesivir	Placebo	Suspended (The epidemic of COVID-19 has been controlled well at present; no eligible patients can be recruited.)	NCT04252664
Comparison of Remdesivir Versus Lopinavir/Ritonavir and Remdesivir Combination in COVID-19 Patients	Ahmed Essam	IV	Remdesivir	Lopinavir/ritonavir + remdesivir	Recruiting	NCT04738045
Efficacy and Safety of DWJ1248 With remdesivir in Severe COVID-19 Patients	Daewoong Pharmaceutical Co. LTD.	III	DWJ1248 + remdesivir	Placebo + remdesivir	Recruiting	NCT04713176
Efficacy and Safety of Remdesivir and Tociluzumab for the Management of Severe COVID-19: A Randomized Controlled Trial	M Abdur Rahim Medical College and Hospital	III	Remdesivir + tocilizumab	N/A	Completed	NCT04678739
REMdesivir-HU Clinical Study and Severe Covid-19 Patients	University of Pecs	III	Remdesivir-HU	N/A	Active, not recruiting	NCT04610541
Study to Evaluate the Safety and Antiviral Activity of Remdesivir (GS-5734™) in Participants With Severe Coronavirus Disease (COVID-19)	Gilead Sciences	III	Remdesivir	N/A	Completed	NCT04292899
Study to Evaluate the Safety and Antiviral Activity of Remdesivir (GS-5734™) in Participants With Moderate Coronavirus Disease (COVID-19) Compared to Standard of Care Treatment	Gilead Sciences	III	Remdesivir	Standard of care	Completed	NCT04292730
Study to Evaluate the Efficacy and Safety of remdesivir in Participants With Severely Reduced Kidney Function Who Are Hospitalized for Coronavirus Disease 2019 (COVID-19) (REDPINE)	Gilead Sciences	III	Remdesivir	Placebo	Recruiting	NCT04745351
Study of Merimepodib in Combination With Remdesivir in Adult Patients With Advanced COVID-19	ViralClear Pharmaceuticals, Inc	II	Merimepodib + remdesivir	Placebo + remdesivir	Terminated (Failure to meet primary endpoint)	NCT04410354
Remdesivir Efficacy in Management of COVID-19 Patients	Ain Shams University	III	Remdesivir	Standard of care	Completed	NCT04853901
A Study to Evaluate the Efficacy and Safety of Remdesivir Plus tocilizumab Compared With remdesivir Plus Placebo in Hospitalized Participants With Severe COVID-19 Pneumonia (REMDACTA)	Hoffmann-La Roche	III	Remdesivir + tocilizumab	Remdesivir + placebo	Completed	NCT04409262
Study to Evaluate the Efficacy and Safety of remdesivir (GS-5734™) Treatment of Coronavirus Disease 2019 (COVID-19) in an Outpatient Setting	Gilead Sciences	III	Remdesivir	Placebo	Active, not recruiting	NCT04501952
Safety, Tolerability and Pharmacokinetics of Inhaled Nanoparticle Formulation of Remdesivir (GS-5734) and NA-831 (Neurosivir)	NeuroActiva, Inc	I	Remdesivir	NA-831; NA-831 + remdesivir	Recruiting	NCT04480333
GS-5734 to Assess the Antiviral Activity, Longer-Term Clearance of Ebola Virus, and Safety in Male Ebola Survivors With Evidence of Ebola Virus Persistence in Semen	National Institute of Allergy and Infectious Diseases (NIAID)	II	Remdesivir	Placebo	Completed	NCT02818582
Factorial Randomized Trial of Rendesivir and Baricitinib Plus Dexamethasone for COVID-19 (the AMMURAVID Trial) (AMMURAVID)	ASST Fatebenefratelli Sacco	III	Remdesivir + dexamethasone	Baricitinib + dexamethasone; remdesivir + baricitinib + dexamethasone	Not yet recruiting	NCT04832880
Treatments for COVID-19: Canadian Arm of the SOLIDARITY Trial (CATCO)	Sunnybrook Health Sciences Centre	II	Remdesivir + standard supportive care	Interferon β-1a + standard supportive care	Recruiting	NCT04330690
Efficacy of Ramdicivir and Baricitinib for the Treatment of Severe COVID 19 Patients	M Abdur Rahim Medical College and Hospital	III	Remdesivir + baricitinib	Remdesivir + tocilizumab	Recruiting	NCT04693026
Adaptive COVID-19 Treatment Trial 2 (ACTT-2)	National Institute of Allergy and Infectious Diseases (NIAID)	III	Remdesivir + baricitinib	Remdesivir + placebo	Completed	NCT04401579
The Efficacy of Different Anti-Viral Drugs in COVID 19 Infected Patients	Oslo University Hospital	II/III	Remdesivir	Hydroxychloroquine	Recruiting	NCT04321616
World Health Organization (WHO) COVID-19 Solidarity Trial for COVID-19 Treatments (SOLIDARITY)	The University of The West Indies	III	Remdesivir	Acalabrutinib; interferon β-1a	Not yet recruiting	NCT04647669
ACTIV-5/Big Effect Trial (BET-A) for the Treatment of COVID-19	National Institute of Allergy and Infectious Diseases (NIAID)	II	Remdesivir + risankizumab	Remdesivir + placebo	Recruiting	NCT04583956
ACTIV-5/Big Effect Trial (BET-B) for the Treatment of COVID-19	National Institute of Allergy and Infectious Diseases (NIAID)	II	Remdesivir + lenzilumab	Remdesivir + placebo	Recruiting	NCT04583969
Adaptive COVID-19 Treatment Trial 4 (ACTT-4)	National Institute of Allergy and Infectious Diseases (NIAID)	III	Remdesivir + baricitinib	Remdesivir + dexamethasone	Active, not recruiting	NCT04640168
National Institute of Allergy and Infectious Diseases (NIAID)	Adaptive COVID-19 Treatment Trial (ACTT)	III	Remdesivir	Placebo	Completed	NCT04280705
An International Randomized Trial of Additional Treatments for COVID-19 in Hospitalized Patients Who Are All Receiving the Local Standard of Care—WHO-SOLIDARITY-GERMANY	Professor Dr. Bernd Mühlbauer	II/III	Remdesivir + standard of care	Standard of care	Active, not recruiting	NCT04575064
Efficacy of favipiravir in Treatment of Mild and Moderate COVID-19 Infection in Nepal	Nepal Health Research Council	III	Favipiravir + placebo	Remdesivir	Recruiting	NCT04694612
Inpatient Treatment With Anti-Coronavirus Immunoglobulin (ITAC)	University of Minnesota	III	Hyperimmune immunoglobulin to SARS-CoV-2 (hIVIG) + remdesivir	Placebo + remdesivir	Active, not recruiting	NCT04546581
Immune Modulators for Treating COVID-19 (ACTIV-1 IM)	Daniel Benjamin	III	Remdesivir + infliximab or matching placebo	Remdesivir + abatacept or matching placebo; remdesivir + cenicriviroc or matching placebo	Recruiting	NCT04593940
COVID-19 and Anti-CD14 Treatment Trial (CaTT)	National Institute of Allergy and Infectious Diseases (NIAID)	II	Remdesivir + anti-CD14	Remdesivir + placebo	Recruiting	NCT04391309
Aralast NP With Antiviral Treatment and Standard of Care Versus Antiviral Treatment With Standard of Care in Hospitalized Patients With Pneumonia and COVID-19 Infection	Blessing Corporate Services, Inc.	III	Remdesivir + alpha1-proteinase inhibitor	Remdesivir	Withdrawn (Administrative Decision)	NCT04675086
First-in-Human Study of Orally Administered GS-441524 for COVID-19	Copycat Sciences LLC	I	Remdesivir	N/A	Active, not recruiting	NCT04859244
Trial to Determine the Efficacy/Safety of Plitidepsin vs Control in Patients With Moderate COVID-19 Infection (Neptuno)	PharmaMar	III	Plitidepsin + dexamethasone	Remdesivir + dexamethasone	Not yet recruiting	NCT04784559
I-SPY COVID-19 TRIAL: An Adaptive Platform Trial for Critically Ill Patients (I-SPY_COVID)	QuantumLeap Healthcare Collaborative	II	Remdesivir	Remdesivir + cenicriviroc; remdesivir + icatibant; remdesivir + pulmozyme; remdesivir + IC14; remdesivir + celecoxib and famotidine; remdesivir + narsoplimab	Recruiting	NCT04488081
ACTIV-3b: Therapeutics for Severely Ill Inpatients With COVID-19 (TESICO)	National Institute of Allergy and Infectious Diseases (NIAID)	III	Aviptadil + remdesivir	Aviptadil + placebo; placebo + remdesivir; placebo	Recruiting	NCT04843761
ACTIV-3: Therapeutics for Inpatients With COVID-19 (TICO)	National Institute of Allergy and Infectious Diseases (NIAID)	III	Remdesivir	LY3819253; VIR-7831; BRII-196/BRII-198; AZD7442; placebo	Recruiting	NCT04501978

(All information in the table are collected from https://clinicaltrials.gov).

### Interferons

The interferons (IFNs) as glycoproteins have broad-spectrum antiviral effects (Lin and Young, 2014). The IFNs can be divided into three types based on the differences in the structures of their respective receptors. In detail, the IFNs are classified into type I IFNs (IFN-α/β), type II IFNs (IFN-γ), and type III IFNs (IFN-λ). Table 4 shows the summary of clinical trials of IFNs on COVID-19 treatment. Mantlo et al. (2020) demonstrated that IFN-α (EC_50_ = 1.35 IU/ml) and IFN-β (EC_50_ = 0.76 IU/ml) at clinically achievable concentrations could suppress the replication of SARC-CoV-2 in Vero cells. These findings provide a valuable fundamental for the potential use of IFN-α/β to against COVID-19. Zhou et al. accessed the efficacy of IFN-α2b and arbidol involving 77 hospitalized patients; as a result, researchers revealed that IFN-α2b with or without arbidol could significantly reduce the duration for detectable virus as well as the inflammatory markers (Zhou Q. et al., 2020). Usually, the IFNs are used in combination with other antiviral therapies (Mantlo et al., 2020). Of note, a group from China examined the effectiveness and safety profile of a triple antiviral therapy including IFN-β1b, lopinavir/ritonavir, and ribavirin with 86 patients suffering from mild to moderate SARS-CoV-2 infection (Hung et al., 2020). Their results showed that the triple combination treatment is superior to lopinavir/ritonavir treatment alone with shorter viral shedding duration and hospital stay period.

**TABLE 4:** Summary of clinical trials of interferons (IFNs) on COVID-19 treatment.

Trial title	Sponsor	Trial phase	Primary intervention	Secondary intervention	Status	Identifier
Double Therapy With IFN-beta 1b and Hydroxychloroquine	The University of Hong Kong	II	Interferon β-1b + hydroxychloroquine	Hydroxychloroquine	Completed	NCT04350281
Pegylated Interferon Lambda Treatment for COVID-19	Raymond Chung	II	Interferon λ	Placebo	Enrolling by invitation	NCT04343976
Pegylated Interferon - α2b With SARSCoV- 2 (COVID-19)	Cadila Healthcare Limited	II	Interferon α-2b	Standard of Care	Recruiting	NCT04480138
Interferon Beta 1a in Hospitalized COVID-19 Patients (IB1aIC)	Shahid Beheshti University of Medical Sciences	IV	Interferon β-1a + lopinavir/ritonavir + single dose of hydroxychloroquine	Lopinavir/ritonavir + single dose of hydroxychloroquine	Enrolling by invitation	NCT04350671
Dual Therapy With Interferon Beta-1b and Clofazimine for COVID-19	The University of Hong Kong	II	Interferon β-1b + clofazimine	Clofazimine	Recruiting	NCT04465695
The Investigation Into Beneficial Effects of High-Dose Interferon Beta 1-a, Compared to Low-dose Interferon Beta 1-a in Moderate to Severe Covid-19	Shahid Beheshti University of Medical Sciences	II	Lopinavir/ritonavir + high dose interferon β-1a	Lopinavir/ritonavir + low dose interferon β-1a	Not yet recruiting	NCT04521400
An Investigation into Beneficial Effects of Interferon Beta 1a, Compared to Interferon Beta 1b and the base Therapeutic Regiment in Moderate to Severe COVID-19: A Randomized Clinical Trial (COVIFERON)	Shahid Beheshti University of Medical Sciences	II	Hydroxychloroquine + lopinavir/ritonavir + interferon β-1a	Hydroxychloroquine + lopinavir/ritonavir + interferon β-1b; hydroxychloroquine + lopinavir/ritonavir	Completed	NCT04343768
Clinical Study for the Treatment With Interferon-ß-1a (IFNß-1a) of COVID-19 Patients (INTERCOP)	Emanuele Bosi	II	Interferon β-1a	Standard care	Terminated (Futility.)	NCT04449380
Treatment of COVID-19 by Nebulization of Interferon Beta 1b Efficiency and Safety Study (COV-NI)	Centre Hospitalier Universitaire, Amiens	II	Type I interferon + routine care	Routine care	Recruiting	NCT04469491
Interferon Lambda for Immediate Antiviral Therapy at Diagnosis in COVID-19 (ILIAD)	University Health Network, Toronto	II	Interferon λ-1a	Placebo	Recruiting	NCT04354259
Pegylated Interferon Lambda for Treatment of COVID-19 Infection	Soroka University Medical Center	II	Interferon λ + standard of care treatment	Standard of care treatment	Recruiting	NCT04534673
IFN-Beta 1b and Remdesivir for COVID19	The University of Hong Kong	II	Interferon β-1b + remdesivir	Remdesivir	Recruiting	NCT04647695
The Containing Coronavirus Disease 19 (COVID-19) Trial (ConCorD-19)	Pontificia Universidad Catolica de Chile	III	Interferon β-1a	Standard of care	Recruiting	NCT04552379
Human Intravenous Interferon Beta-Ia Safety and Preliminary Efficacy in Hospitalized Subjects With CoronavirUS (HIBISCUS)	Faron Pharmaceuticals Ltd.	II	Interferon β-1a	Placebo	Not yet recruiting	NCT04860518
Rintatolimod and IFN Alpha-2b for the Treatment of Mild or Moderate COVID-19 Infection in Cancer Patients	Roswell Park Cancer Institute	I/II	Recombinant interferon α-2b + rintatolimod	Rintatolimod	Recruiting	NCT04379518
IFN Beta-1b and Ribavirin for Covid-19	The University of Hong Kong	II	Interferon β-1b + ribavirin + standard care	Standard care	Recruiting	NCT04494399
Adaptive COVID-19 Treatment Trial 3 (ACTT-3)	National Institute of Allergy and Infectious Diseases (NIAID)	III	Remdesivir + interferon β-1a	Remdesivir + placebo	Completed	NCT04492475
Interferon Lambda Therapy for COVID-19	Icahn School of Medicine at Mount Sinai	II	Interferon λ-1a	Supportive care	Withdrawn (Due to the number of competing trials at their site, the study team has closed enrollment and withdrawn this trial.)	NCT04388709
Evaluation of Ganovo (Danoprevir) Combined With Ritonavir in the Treatment of SARS-CoV-2 Infection	The Ninth Hospital of Nanchang	IV	Ganovo + ritonavir + interferon nebulization	Ganovo + ritonavir	Completed	NCT04291729
COVID-19 Prevention and Treatment in Cancer; a Sequential Multiple Assignment Randomized Trial; (C-SMART)	Peter MacCallum Cancer Centre, Australia	III	Interferon α	Selinexor; lenzilumab	Recruiting	NCT04534725
An Open-Label Study to Assess Response to COVID-19 Vaccine in Multiple Sclerosis Participants Treated With ofatumumab	Novartis Pharmaceuticals	IV	Ofatumumab + mRNA COVID-19 vaccine	Interferon or glatiramer acetate + mRNA COVID-19 vaccine	Not yet recruiting	NCT04878211
Trial of COVID-19 Outpatient Treatment in Individuals With Risk Factors for Aggravation (COVERAGEFrance)	University Hospital, Bordeaux	II/III	Interferon β-1b	Telmisartan; ciclesonide; vitamins	Recruiting	NCT04356495
Anti-Coronavirus Therapies to Prevent Progression of Coronavirus Disease 2019 (COVID-19) Trial (ACTCOVID19)	Population Health Research Institute	III	Interferon β	Colchicine; aspirin; rivaroxaban; usual care	Recruiting	NCT04324463
Peginterferon Lambda-1a for the Prevention and Treatment of SARS-CoV-2 (COVID-19) Infection (PROTECT)	Johns Hopkins University	II	Interferon λ-1a	Placebo	Terminated (Single left study with low enrollment)	NCT04344600

(All information in the table are collected from https://clinicaltrials.gov).

However, some reports indicated that the application of IFN-λ have more advantages in COVID-19 treatment. The most outstanding profile of IFN-λ over IFN-α/β is the absence of pro-inflammatory effects (Prokunina-Olsson et al., 2020). This is because the response to IFN-λ administration localizes to epithelial cells, which could reduce side effects and inflammatory effects related to the systemic action from IFN-α/β treatment. Also, researchers showed that IFN-λ reduces the presence of virus in the lungs and prevents the induction of cytokine storm; hence, the application of IFN-λ could avoid pneumonia and acute respiratory distress syndrome (ARDS) (Andreakos and Tsiodras, 2020). Overall, IFN-λ is a promising and potential therapeutic agent for patients suffering from COVID-19. Notably, more clinical study is necessary in the future.

### Umifenovir

Umifenovir (brand name: Arbidol, ARB) is an antiviral drug, which has the ability to inhibit the replication of influenza A and B virus through impeding the early membrane fusion event (Leneva et al., 2009). Table 5 indicates the summary of clinical trials of umifenovir for the treatment of COVID-19. Zhu et al. (2020) accessed the efficacy and safety of lopinavir/ritonavir and umifenovir involving 50 COVID-19 patients, 34 cases with lopinavir/ritonavir treatment, and 16 cases with umifenovir treatment. From the results, no side effects and developed pneumonia or ARDS were observed in both groups. More importantly, patients with umifenovir treatment have shorter duration of positive RNA test compared with those with lopinavir/ritonavir treatment; thus, the authors indicated that umifenovir may be superior to lopinavir/ritonavir against COVID-19. Similarly, Deng et al. (2020) demonstrated that lopinavir/ritonavir combined with umifenovir had more favorable clinical outcomes compared with lopinavir/ritonavir only in a retrospective cohort study. Furthermore, Nojomi et al. (2020) evaluated HCQ followed by lopinavir/ritonavir or HCQ followed by umifenovir among 100 patients with COVID-19. As a result, the researchers found that patients in the umifenovir group had shorter hospitalized duration and higher peripheral oxygen saturation rate, also had improvements in requiring ICU admissions, and chest CT involvement. Moreover, some studies showed that umifenovir was well-tolerated with mild gastrointestinal tract reaction and related to the lower mortality in COVID-19 cases (Jomah et al., 2020).

**TABLE 5:** Summary of clinical trials of umifenovir on COVID-19 treatment.

Trial title	Sponsor	Trial phase	Primary intervention	Secondary intervention	Status	Identifier
Study of Efficacy and Safety of TL-FVP-t vs. SOC in Patients With Mild to Moderate COVID-19	R-Pharm	III	Favipiravir + stand of care	Umifenovir + intranasal recombinant interferon α, or hydroxychloroquine, or chloroquine, or mefloquine in recommended regimen	Active, not recruiting	NCT04501783
Umifenovir in Hospitalized COVID-19 Patients (UAIIC)	Shahid Beheshti University of Medical Sciences	IV	Umifenovir + interferon β-1a + lopinavir/ritonavir + single dose of hydroxychloroquine + standards of care	Interferon β-1a + lopinavir/ritonavir + single dose of hydroxychloroquine + standards of care	Enrolling by invitation	NCT04350684

(All information in the table are collected from *https://clinicaltrials.gov* ).

However, Lian et al. (2020) indicated that umifenovir is not relative to the improved response in non-ICU COVID-19 patients in a retrospective study. In detail, the study included 81 patients suffering from COVID-19, and evaluated several baseline clinical and laboratory factors. Of note, the patients with umifenovir treatment even had longer hospital stay duration than those patients in the control group. Hence, the authors indicated that umifenovir might not improve prognosis or accelerate SARS-CoV-2 clearance in non-ICU patients with COVID-19.

### Azithromycin

Azithromycin is a macrolide antibiotic medication. Azithromycin binds to the 50S subunit of ribosome, and thereby prevents the mRNA translation and interferes with protein synthesis (Bakheit et al., 2014). Table 6 summarizes the clinical trials of azithromycin on COVID-19 treatment. Gautret et al. (2020) showed that azithromycin could reinforce the effectiveness of HCQ to clear the COVID-19 virus. Of note, the sample size was small, which only involved 20 cases. Also, researchers revealed that azithromycin combined with HCQ, or with lopinavir/ritonavir, could improve the clinical response and accelerate the COVID-19 virus clearance (Purwati et al., 2021). By contrast, Cavalcanti et al. (2020) reported that no improved clinical outcomes were observed in COVID-19 patients, suffering from mild to moderate COVID-19, treated with HCQ alone or with azithromycin compared with those with standard care in a multicenter, randomized, open-label, three-group, controlled trial involving 667 patients. Also, evidence from retrospective observational studies demonstrated that azithromycin in combination with HCQ did not induce favorable clinical outcomes for COVID-19 patients (Echeverría-Esnal et al., 2021).

**TABLE 6:** Summary of clinical trials of azithromycin on COVID-19 treatment.

Trial title	Sponsor	Trial phase	Primary intervention	Secondary intervention	Status	Identifier
Hydroxychloroquine Plus Azithromycin Versus Hydroxychloroquine for COVID-19 Pneumonia (COVIDOC Trial) (COVIDOC)	University Hospital, Montpellier	II/III	Hydroxychloroquine + azithromycin	Azithromycin	Terminated (Halted prematurely.)	NCT04345861
Study of Immune Modulatory Drugs and Other Treatments in COVID-19 Patients: Sarilumab, Azithromycin, Hydroxychloroquine Trial—CORIMUNO-19-VIRO (CORIMUNO-VIRO)	Assistance Publique - Hôpitaux de Paris	II/III	Sarilumab + azithromycin + hydroxychloroquine	Sarilumab	Suspended (DSMB recommendation (futility).)	NCT04341870
A Multileft Open-Label Two-arm Randomized Superiority Clinical Trial of Azithromycin Versus Usual Care in Ambulatory COVID19 (ATOMIC2) (ATOMIC2)	University of Oxford	III	Azithromycin	N/A	Completed	NCT04381962
Hydroxychloroquine Azithromycin COVID-19 Pregnancy Trial (HASCOPT)	Hospital St. Joseph, Marseille, France	III	Hydroxychloroquine + azithromycin	Conventional management	Withdrawn (No authorization obtained.)	NCT04365231
HOPE: A Trial of Hydroxychloroquine Plus Azithromycin in High Risk COVID-19 (HOPE_BRAZIL)	Latin American Cooperative Oncology Group	II	Hydroxychloroquine + azithromycin	Hydroxychloroquine + placebo	Withdrawn (Withdrawn due to lack of study lefts interested in participating.)	NCT04575558
Azithromycin for COVID-19 Treatment in Outpatients Nationwide (ACTION)	Thomas M. Lietman	III	Azithromycin	Placebo	Active, not recruiting	NCT04332107
Atovaquone and Azithromycin Combination for Confirmed COVID-19 Infection	HonorHealth Research Institute	II	Atovaquone/Azithromycin	N/A	Recruiting	NCT04339426
Proactive Care of Ambulatory COVID19 Patients (AMBU-COVID)	Centre Hospitalier Universitaire, Amiens	III	Azithromycin	Symptomatic treatment	Not yet recruiting	NCT04371107
Hydroxychloroquine vs. Azithromycin for Outpatients in Utah With COVID-19 (HyAzOUT)	Intermountain Health Care, Inc	III	Hydroxychloroquine	Azithromycin	Recruiting	NCT04334382
Hydroxychloroquine vs. Azithromycin for Hospitalized Patients With Suspected or Confirmed COVID-19 (HAHPS)	Intermountain Health Care, Inc	II	Hydroxychloroquine	Azithromycin	Active, not recruiting	NCT04329832
Open Label Non-Comparative Trial of the Combination of Hydroxychloroquine and Azithromycin in the Treatment of Hospitalized Patients	University of New Mexico	II	Hydroxychloroquine + azithromycin	N/A	Active, not recruiting	NCT04458948
Randomized Comparison of Combination Azithromycin and Hydroxychloroquine vs. Hydroxychloroquine Alone for the Treatment of Confirmed COVID-19	Rutgers, The State University of New Jersey	II	Hydroxychloroquine sulfate + azithromycin	Hydroxychloroquine sulfate	Active, not recruiting	NCT04336332
Azithromycin + Amoxicillin/Clavulanate vs Amoxicillin/Clavulanate in COVID19 Patients with Pneumonia in Non-intensive Unit (AziA)	Nantes University Hospital	III	Azithromycin + amoxicillin/clavulanate	Amoxicillin/clavulanate	Not yet recruiting	NCT04363060
Safety and Efficacy of Hydroxychloroquine Associated with Azithromycin in SARS-Cov-2 Virus (COVID-19) (Coalition-I)	Hospital do Coracao	III	Hydroxychloroquine	Hydroxychloroquine + azithromycin	Active, not recruiting	NCT04322123
Hydroxychloroquine, Azithromycin in the Treatment of Covid-19 (PACTT)	Centre Hôpital Universitaire Farhat Hached	III	Hydroxychloroquine + azithromycin	Hydroxychloroquine + placebo	Not yet recruiting	NCT04405921
Evaluation of Prognostic Modification in COVID-19 Patients in Early Intervention Treatment	Gilberto Cruz arteaga	III	Azithromycin + ivermectin + ribaroxaban + paracetamol	Azithromycin + ribaroxaban + paracetamol	Recruiting	NCT04673214
Evaluating the Efficacy of Hydroxychloroquine and Azithromycin to Prevent Hospitalization or Death in Persons With COVID-19	National Institute of Allergy and Infectious Diseases (NIAID)	II	Hydroxychloroquine + azithromycin	Placebo	Terminated (Slow enrollment and lack of community enthusiasm.)	NCT04358068
Hydroxychloroquine Monotherapy and in Combination with Azithromycin in Patients With Moderate and Severe COVID-19 Disease	Novartis Pharmaceuticals	III	Hydroxychloroquine	Hydroxychloroquine + azithromycin	Completed	NCT04358081
A Single-blinded, Randomized, Placebo Controlled phase II Trial of Prophylactic Treatment With Oral Azithromycin Versus Placebo in Cancer Patients Undergoing Antineoplastic Treatment During the Corona Virus Disease 19 (COVID-19) Pandemic	Prof. Dr. Matthias Preusser	II	Azithromycin	Placebo	Recruiting	NCT04369365
Efficacy of Hydroxychloroquine, Telmisartan and Azithromycin on the Survival of Hospitalized Elderly Patients With COVID-19 (COVID-Aging)	University Hospital, Strasbourg, France	III	Hydroxychloroquine	Azithromycin; telmisartan	Recruiting	NCT04359953
Proactive Protection With Azithromycin and Hydroxychloroquine in Hospitalized Patients With COVID-19 (ProPAC-COVID)	Chronic Obstructive Pulmonary Disease Trial Network, Denmark	II	Hydroxychloroquine + azithromycin	Placebo	Completed	NCT04322396
Azithromycin Added to Hydroxychloroquine in Patients Admitted to Intensive Care With COVID-19: Randomized Controlled Trial (AZIQUINE-ICU)	Frantisek Duska, MD, PhD	III	Hydroxychloroquine + azithromycin	Hydroxychloroquine	Terminated (Steering Committee decision in accordance with stopping rule 1: Emergence of new data.)	NCT04339816
Open Label Study to Compare Efficacy, Safety and Tolerability of Hydroxychloroquine Combined With Azithromycin Compared to Hydroxychloroquine Combined With Camostat Mesylate and to “No Treatment” in SARS CoV 2 Virus (COSTA)	Sheba Medical Center	III	Hydroxychloroquine + azithromycin	Hydroxychloroquine + camostat mesylate	Recruiting	NCT04355052
Asymptomatic COVID-19 Trial (ACT)	Rutgers, The State University of New Jersey	II	Hydroxychloroquine + azithromycin	Placebo	Withdrawn (The investigators have decided not to go forward with this protocol.)	NCT04374552
Hydroxychloroquine and Zinc With Either Azithromycin or Doxycycline for Treatment of COVID-19 in Outpatient Setting	St. Francis Hospital, New York	IV	Hydroxychloroquine + azithromycin + zinc sulfate	Hydroxychloroquine + doxycycline + zinc sulfate	Completed	NCT04370782
Epidemiology of SARS-CoV-2 and Mortality to Covid19 Disease in French Cancer Patients (ONCOVID)	Gustave Roussy, Cancer Campus, Grand Paris	II	Hydroxychloroquine + azithromycin	Hydroxychloroquine	Recruiting	NCT04341207
Use of Hydroxychloroquine Alone or Associated for Inpatients With SARS-CoV2 Virus (COVID-19)	Apsen Farmaceutica S.A.	III	Hydroxychloroquine sulfate	Hydroxychloroquine sulfate + azithromycin	Withdrawn (This study was canceled before enrollment due to a decision by the Sponsor.)	NCT04361461
Phytomedicines Versus Hydroxychloroquine as an Add On Therapy to Azithromycin in Asymptomatic Covid-19 Patients (PHYTCOVID-19)	Institute for Research and Development of Medicinal and Food Plants of Guinea	II	Hydroxychloroquine + azithromycin	Quinine + azithromycin	Enrolling by invitation	NCT04501965
Study Evaluating the Efficacy of Hydroxychloroquine and Azithromycin in Patients With COVID-19 and Hematological Malignancies (HYACINTHE) (HYACINTHE)	Institut de cancérologie Strasbourg Europe	II	Hydroxychloroquine + azithromycin	Placebo	Withdrawn (Competent authority decision.)	NCT04392128
Hydroxychloroquine, Oseltamivir and Azithromycin for the Treatment of COVID-19 Infection: An RCT (PROTECT)	Shehnoor Azhar	III	Azithromycin	Hydroxychloroquine; oseltamivir; hydroxychloroquine + azithromycin; hydroxychloroquine + oseltamivir; oseltamivir + azithromycin; hydroxychloroquine + oseltamivir + azithromycin	Recruiting	NCT04338698
Efficacy of Sofosbuvir Plus Ledipasvir in Egyptian Patients With COVID-19 Compared to Standard Treatment	Almaza Military Fever Hospital	III	Sofosbuvir + ledipasvir	Oseltamivir + hydroxychloroquine and azithromycin	Completed	NCT04530422
International ALLIANCE Study of Therapies to Prevent Progression of COVID-19	National Institute of Integrative Medicine, Australia	II	Vitamin C + hydroxychloroquine + azithromycin + zinc citrate + vitamin D3 + vitamin B12	Hydroxychloroquine + azithromycin + zinc citrate + vitamin D3 + vitamin B12	Recruiting	NCT04395768
VA Remote and Equitable Access to COVID-19 Healthcare Delivery (VA-REACH TRIAL) (VA-REACH)	Salomeh Keyhani MD	III	Hydroxychloroquine	Azithromycin	Suspended (Concerns related to study drug)	NCT04363203
Evaluate the Efficacy and Safety of Oral Hydroxychloroquine, Indomethacin and Zithromax in Subjects With Mild Symptoms of COVID-19 (COVID-19)	Perseverance Research Center, LLC	I/II	Hydroxychloroquine	Azithromycin; indomethacin	Recruiting	NCT04344457
Effectiveness of Hydroxychloroquine in COVID-19 Patients (COVID)	Prof. Dr. Umar Farooq	III	Hydroxychloroquine	Azithromycin	Not yet recruiting	NCT04328272
Levamisole and Isoprinosine in the Treatment of COVID19: A Proposed Therapeutic Trial	Cairo University	III	Levamisole + isoprinosine	Azithromycin + hydroxychloroquine	Not yet recruiting	NCT04383717
Efficacy and Safety of Favipiravir in COVID-19 Patients in Indonesia (FVR)	Ina-Respond	III	Favipiravir + azithromycin	Favipiravir	Suspended (Study halted prematurely but potentially will resume, the protocol will be amended.)	NCT04613271
Treatment in Patients With Suspected or Confirmed COVID-19 With Early Moderate or Severe Disease (RCT)	LCMC Health	III	Hydroxychloroquine	Azithromycin	Active, not recruiting	NCT04344444
Safety and Efficacy of Doxycycline and Rivaroxaban in COVID-19 (DOXYCOV)	Yaounde Central Hospital	IV	Doxycycline + Rivaroxaban	Hydroxychloroquine + azithromycin	Recruiting	NCT04715295
A Study of Quintuple Therapy to Treat COVID-19 Infection (HAZDpaC)	ProgenaBiome	II	Hydroxychloroquine + azithromycin + vitamin C + vitamin D + zinc	Vitamin C + vitamin D + zinc	Recruiting	NCT04334512
Safety and Efficacy of Hydroxychloroquine for the Treatment and Prevention of Coronavirus Disease 2019 (COVID-19) Caused by Severe Acute Respiratory Syndrome Coronavirus 2 (SARS-CoV-2)	International Brain Research Foundation	I	Hydroxychloroquine + azithromycin + vitamins and minerals	N/A	Not yet recruiting	NCT04590274
Effectiveness of Ivermectin as Add-On Therapy in COVID-19 Management	University of Baghdad	I	Ivermectin + hydroxychloroquine + azithromycin	N/A	Completed	NCT04343092
Ivermectin for Severe COVID-19 Management	Afyonkarahisar Health Sciences University	III	Ivermectin	Hydroxychloroquine, favipiravir and azithromycin	Completed	NCT04646109
Assessment of Efficacy and Safety of HCQ and Antibiotics Administrated to Patients COVID19(+) (COVID + PA)	Abderrahmane Mami Hospital	IV	Hydroxychloroquine + azithromycin	N/A	Withdrawn (- Interest in the use of HCQ is controversial.)	NCT04351919
Evaluation of the Efficacy and Safety of Treatments for Patients Hospitalized for COVID-19 Infection Without Signs of Acute Respiratory Failure, in Tunisia (THINC)	Abderrahmane Mami Hospital	III	Hydroxychloroquine + azithromycin	Hydroxychloroquine + azithromycin + zinc; azithromycin + doxycycline	Withdrawn (- Interest in the use of HCQ is controversial.)	NCT04528927
Administration of Allogenic UC-MSCs as Adjuvant Therapy for Critically Ill COVID-19 Patients	Indonesia University	I	Oseltamivir + azithromycin	Oseltamivir + azithromycin + umbilical-cord derived mesenchymal stem cells	Recruiting	NCT04457609
OUTpatient Treatment of COVID-19 in Patients With Risk Factor for Poor Outcome (OUTCOV)	Groupe Hospitalier Paris Saint Joseph	III	Azithromycin	Hydroxychloroquine; lopinavir/ritonavir	Withdrawn (The PI decided.)	NCT04365582
A Study of the Effectiveness of an Off Label Mefloquine Use for the Treatment of Patients With COVID19	Burnasyan Federal Medical Biophysical Center	II/III	Mefloquine	Hydroxychloroquine; mefloquine + azithromycin ± tocilizumab; hydroxychloroquine + azithromycin ± tocilizumab	Completed	NCT04347031
Add on to Azithromycin, Phytomedicine and/or Antimalarial Drug vs Hydroxychloroquine in Uncomplicated COVID-19 Patients (CANCOVID-19)	Institute for Research and Development of Medicinal and Food Plants of Guinea	II	Hydroxychloroquine + azithromycin	Cospherunate + azithromycin; cospherunate + phytomedicine + azithromycin	Enrolling by invitation	NCT04502342
Efficacy and Safety of Hydroxychloroquine and Favipiravir in the Treatment of Mild to Moderate COVID-19	Ministry of Health, Turkey	III	Favipiravir	Favipiravir + hydroxychloroquine; favipiravir + azithromycin; hydroxychloroquine + azithromycin	Active, not recruiting	NCT04411433
Efficacy and Safety Evaluation of Treatment Regimens in Adult COVID-19 Patients in Senegal (SEN-CoV-Fadj)	Institut Pasteur de Dakar	III	Hydroxychloroquine + azithromycin	Hydroxychloroquine + azithromycin + nafamostat mesilate	Recruiting	NCT04390594

(All information in the table are collected from https://clinicaltrials.gov).

### Antibacterial/antibiotic drugs

It has been reported that bacterial coinfection happened in 3.5% of COVID-19 patients (Sieswerda et al., 2021). In other words, the hospitalized patients with COVID-19 have risk of bacterial infections. Sieswerda et al. (2021) recommended that the 5-day antibiotic therapy is required for the COVID-19 patients suffering with suspected bacterial respiratory infection after clinical improvements. However, their recommendation needs to be confirmed because unnecessary antibiotic treatment should be prevented. Also, some studies revealed that bacterial and fungal coinfection would occur in patients with SARS-CoV-2 infection, thereby the antimicrobial treatment regimen and stewardship interventions are necessary to control the exacerbating COVID-19 pandemic (Rawson et al., 2020). More importantly, antimicrobial resistance should be considered as the collateral effect of SARS-CoV-2 infection, and thus, proper trend for antibiotic stewardship interventions should be analyzed and prescribed in the emergency department (Pulia et al., 2020).

## Immunotherapy

### Monoclonal antibody

#### Tocilizumab

Tocilizumab (TCZ, trade name: Actemra) is a recombinant humanized monoclonal antibody (Sheppard et al., 2017). TCZ is well-tolerated without significant abnormalities after long-term toxicity tests on animals (Gabay et al., 2013). For the mechanism of action, TCZ specially binds membrane-bound interleukin-6 receptor (mIL-6R) and soluble interleukin-6 receptor (sIL-6R) and inhibits signal transduction (Ibrahim et al., 2020). It has been reported that COVID-19 induces higher plasma levels of cytokines including, but not limited to, IL-6, IL-2, IL-7, IL-10, tumor necrosis factor-α (TNF-α), IFN-γ-inducible protein, etc., in ICU patients with SARS-CoV-2 infection (Chen N. et al., 2020; Huang C. et al., 2020), which refers to a cytokine storm in patients. Furthermore, several studies indicated that TCZ treatment could return the temperature to normal quickly and improve the respiratory function through blocking IL-6 receptors (Fu et al., 2020; Zhang et al., 2020). Table 7 shows the clinical trials of TCZ on COVID-19 treatment. Luo et al. (2020) examined the efficacy of TCZ, as a recombinant humanized antihuman IL-6 receptor monoclonal antibody, and found that the serum IL-6 level decreased in 10 patients, while the persistent and dramatic increase in IL-6 was found in four patients who failed in the treatment. In contrast, Xu et al. (2020) recorded the clinical manifestation, computerized tomography (CT) scan image, and laboratory examinations to assess the effectiveness of TCZ in severe COVID-19 patients. Their results showed that TCZ has critical roles in pathogenesis and clinical improvement in patients. Moreover, Klopfenstein et al. (2020) performed a retrospective case-control study involving 20 patients with severe SARS-CoV-2 infection and found that TCZ could reduce the number of ICU admissions and/or mortality compared with the patients without TCZ therapy. It should be noticed that the study performed by Klopfenstein et al. has some limitations, such as the small sample size and the retrospective nature of their work.

**TABLE 7:** Summary of clinical trials of tocilizumab (TCZ) on COVID-19 treatment.

Trial title	Sponsor	Trial phase	Primary intervention	Secondary intervention	Status	Identifier
Tocilizumab in Coronavirus-19 Positive Patients	University of Calgary	III	Tocilizumab	N/A	Not yet recruiting	NCT04423042
Efficacy of Tocilizumab on Patients With COVID-19	Massachusetts General Hospital	III	Tocilizumab	Placebo	Completed	NCT04356937
Tocilizumab in COVID-19 Lahore General Hospital (TC19LGH)	Lahore General Hospital	I	Tocilizumab	N/A	Recruiting	NCT04560205
Clinical Trial to Evaluate the Effectiveness and Safety of Tocilizumab for Treating Patients With COVID-19 Pneumonia	Fundacion SEIMC-GESIDA	II	Tocilizumab	N/A	Completed	NCT04445272
Tocilizumab - An Option for Patients With COVID-19 Associated Cytokine Release Syndrome; A Single Center Experience	FMH College of Medicine and Dentistry	IV	Tocilizumab	Methylprednisolone	Completed	NCT04730323
Safety and Efficacy of tocilizumab in Moderate to Severe COVID-19 With Inflammatory Markers (TOCIBRAS)	Beneficência Portuguesa de São Paulo	III	Tocilizumab + supportive care	Supportive care	Terminated (Safety)	NCT04403685
Low-Dose Tocilizumab Versus Standard of Care in Hospitalized Patients With COVID-19 (COVIDOSE-2)	University of Chicago	II	Tocilizumab	Standard of care	Recruiting	NCT04479358
Tocilizumab for SARS-CoV2 (COVID-19) Severe Pneumonitis	Università Politecnica delle Marche	II	Tocilizumab	N/A	Active, not recruiting	NCT04315480
Efficacy of Tocilizumab in Modifying the Inflammatory Parameters of Patients With COVID-19 (COVITOZ-01) (COVITOZ-01)	Hospital Universitario Ramon y Cajal	II	Tocilizumab	Standard care	Recruiting	NCT04435717
Tocilizumab in the Treatment of Coronavirus Induced Disease (COVID-19) (CORON-ACT)	University Hospital Inselspital, Berne	II	Tocilizumab	Placebo	Terminated (1.) Not possible to recruit the planned number of patients during the planned study period; 2.) “Dexamethasone” was included in the standard of care for the study population during the course of the study and inclusion criteria could no longer be met.)	NCT04335071
Tocilizumab for Patients With Cancer and COVID-19 Disease	National Cancer Institute (NCI)	II	Tocilizumab	N/A	Terminated (Other - Randomized data no longer support continuation.)	NCT04370834
Evaluating Tocilizumab for Severe COVID-19 Infection in Breast Cancer vs. Non-Cancer Pateints	Beni-Suef University	II	Tocilizumab	N/A	Recruiting	NCT04871854
Tocilizumab in COVID-19 Pneumonia (TOCIVID-19) (TOCIVID-19)	National Cancer Institute, Naples	II	Tocilizumab	N/A	Active, not recruiting	NCT04317092
Tocilizumab Treatment in Patients With COVID-19	Instituto Nacional de Cancerologia de Mexico	II	Tocilizumab	N/A	Active, not recruiting	NCT04363853
Trial of Tocilizumab for Treatment of Severe COVID-19: ARCHITECTS (ARCHITECTS)	Queen’s Medical Centre	III	Tocilizumab	Placebo	Recruiting	NCT04412772
Tocilizumab to Prevent Clinical Decompensation in Hospitalized, Non-Critically Ill Patients With COVID-19 Pneumonitis (COVIDOSE)	University of Chicago	II	Tocilizumab	N/A	Completed	NCT04331795
Assessment of Efficacy and Safety of Tocilizumab Compared to DefeROxamine, Associated With Standards Treatments in COVID-19 (+) Patients Hospitalized in Intensive Care in Tunisia (TRONCHER)	Abderrahmane Mami Hospital	III	Tocilizumab	Deferoxamine	Not yet recruiting	NCT04361032
Comparison of Tocilizumab Plus Dexamethasone vs. Dexamethasone for Patients With Covid-19 (TOCIDEX)	Assistance Publique - Hôpitaux de Paris	II	Dexamethasone + tocilizumab	Dexamethasone	Recruiting	NCT04476979
Tocilizumab Versus Methylprednisolone in the Cytokine Release Syndrome of Patients With COVID-19	Hospital Sao Domingos	II	Tocilizumab	Methylprednisolone	Not yet recruiting	NCT04377503
Clinical Trial of the Use of tocilizumab for Treatment of SARS-CoV-2 Infection (COVID-19) (TOCOVID)	Fundació Institut de Recerca de l'Hospital de la Santa Creu i Sant Pau	II	Tocilizumab + hydroxychloroquine + azithromycin	Hydroxychloroquine + azithromycin	Recruiting	NCT04332094
Tocilizumab for Prevention of Respiratory Failure in Patients With Severe COVID-19 Infection	Memorial Sloan Kettering Cancer Center	II	Tocilizumab	N/A	Active, not recruiting	NCT04377659
Clinical Efficacy of Heparin and Tocilizumab in Patients With Severe COVID-19 Infection (hepmab)	University of Sao Paulo	III	Heparin + tocilizumab	Heparin	Recruiting	NCT04600141
Study to Evaluate the Efficacy and Safety of Tocilizumab Versus Corticosteroids in Hospitalized COVID-19 Patients With High Risk of Progression	University of Malaya	III	Tocilizumab	Methylprednisolone	Not yet recruiting	NCT04345445
Efficacy of Early Administration of Tocilizumab in COVID-19 Patients	Azienda Unità Sanitaria Locale Reggio Emilia	II	Tocilizumab	Standard of care	Terminated (Based on interim analysis for futility and given an enrollment rate almost nil.)	NCT04346355
The Use of Tocilizumab in the Management of Patients Who Have Severe COVID-19 With Suspected Pulmonary Hyperinflammation	Hadassah Medical Organization	IV	Tocilizumab	Placebo	Recruiting	NCT04377750
A Study in Patients With COVID-19 and Respiratory Distress Not Requiring Mechanical Ventilation, to Compare Standard-of-Care With Anakinra and Tocilizumab Treatment the Immunomodulation-CoV Assessment (ImmCoVA) Study	Karolinska University Hospital	II	Tocilizumab + standard of care	Anakinra + standard of care	Recruiting	NCT04412291
A Study to Evaluate the Safety and Efficacy of Tocilizumab in Patients With Severe COVID-19 Pneumonia (COVACTA)	Hoffmann-La Roche	III	Tocilizumab	Placebo	Completed	NCT04320615
A Study to Investigate Intravenous Tocilizumab in Participants With Moderate to Severe COVID-19 Pneumonia (MARIPOSA)	Hoffmann-La Roche	II	Tocilizumab	N/A	Completed	NCT04363736
A Study to Evaluate the Efficacy and Safety of Tocilizumab in Hospitalized Participants With COVID-19 Pneumonia (EMPACTA)	Genentech, Inc	III	Tocilizumab	Placebo	Recruiting	NCT04372186
COVID-19: Salvage Tocilizumab as a Rescue Measure (COVIDSTORM)	Jarmo Oksi	III	Tocilizumab	Standard of care	Recruiting	NCT04577534
A Trial Using Anakinra, Tocilizumab Alone or in Association With Ruxolitinib in Severe Stage 2b and 3 of COVID19-Associated Disease (INFLAMMACOV)	Assistance Publique Hopitaux De Marseille	III	Tocilizumab ± ruxolitinib	Anakinra ± ruxolitinib	Not yet recruiting	NCT04424056
Tocilizumab for the Treatment of Cytokine Release Syndrome in Patients With COVID-19 (SARS-CoV-2 Infection)	Emory University	III	Tocilizumab + standard of care	Standard of care	Withdrawn (Study abandoned due to drug billing issues.)	NCT04361552
Checkpoint Blockade in COVID-19 Pandemic (COPERNICO)	MedSIR	II	Tocilizumab + pembrolizumab	Standard of care	Recruiting	NCT04335305
Personalized Immunotherapy for SARS-CoV-2 (COVID-19) Associated With Organ Dysfunction (ESCAPE)	Hellenic Institute for the Study of Sepsis	II	Tocilizumab	Anakinra	Completed	NCT04339712
Treatment of COVID-19 Patients With Anti-Interleukin Drugs (COV-AID)	University Hospital, Ghent	III	Tocilizumab	Anakinra + tocilizumab; anakinra; anakinra + siltuximab; usual care	Active, not recruiting	NCT04330638

(All information in the table are collected from https://clinicaltrials.gov).

Interestingly, Stone et al. (2020) conducted a randomized, double-blind, placebo-controlled study (ClinicalTrials.gov, NCT04356937) with a larger sample size (243 patients with severe SARS-CoV-2 infection). The results from the study of Stone et al. demonstrated that TCZ is not effective in preventing intubation or death. However, some benefits, such as fewer serious infections in patients receiving TCZ therapy, cannot be ignored. Most recently, Salama et al. (2021) performed a trial enrolled with 389 COVID-19 patients (ClinicalTrials.gov, NCT04372186). The results showed that TCZ cannot improve survival rate; it only reduced the possibility of progression to the composite outcome of mechanical ventilation or death for the patients who were not receiving mechanical ventilation. Currently, TCZ undergoes several phase III clinical trials (Clinicaltrials.gov, NCT04423042, NCT04356937, NCT04403685, etc.) to further understand the TCZ treatment as a supportive care option in alleviating the severe respiratory symptoms correlated with SARS-CoV-2 infection (Alzghari and Acuña, 2020). Overall, TCZ appears to be an effective treatment for COVID-19 patients to calm the inflammatory storm and to reduce mortality. Notably, the efficacy of TCZ is controversial and remains to be further determined.

#### Mepolizumab

Mepolizumab (brand name: Nucala) is a human monoclonal antibody medication used for the treatment of severe eosinophilic asthma, eosinophilic granulomatosis, and hypereosinophilic syndrome (HES) (Mukherjee et al., 2014; Ennis et al., 2019). Mepolizumab binds to IL-5 and prevents it from binding to its receptor on the surface of eosinophil white blood cells. Notably, some experts recommended to continue the mepolizumab therapy in COVID-19 patients with severe eosinophilic asthma, but the concern is that eosinopenia, which may serve as a diagnostic indicator for COVID-19 disease, may be a risk factor for worse disease outcomes (Li Q. et al., 2020; Du et al., 2020; Bousquet et al., 2021). In other words, it is a challenge to manage patients with severe eosinophilic asthma infected by SARS-CoV-2. Aksu et al. (2021) reported that no evidence of loss of asthma control was observed during mepolizumab therapy in a woman patient with asthma infected by SARS-CoV-2. In addition, Azim et al. (2021) observed the outcomes from four patients receiving mepolizumab treatment. The researchers found that all four patients had a further reduction in their eosinophil counts within the reference range at the presentation with SARS-CoV-2 infection, but the underlying mechanism is not fully investigated, and subsequently recovered without any immediate evidence of long-term respiratory outcomes. Of note, one of four patients required hospitalization and ventilatory support. They thereby suggested that the mepolizumab therapy should be continued without any changed outcomes in the COVID-19 course. However, evidence from Eger et al. (2020) involved 634 severe asthma patients diagnosed with COVID-19 showed that patients with severe asthma receiving mepolizumab therapy have a more severe course of COVID-19 and an increasing risk of severity of COVID-19 compared with the general population. Overall, because the relevant data are limited, and the guideline is currently absent, maintaining or postponing mepolizumab treatment until the patient recovers from SARS-CoV-2 infection should be a case-by-case based decision for COVID-19 patients with severe asthma.

#### Sarilumab

Sarilumab (brand name: Kevzara) is a humanized monoclonal antibody against IL-6 receptor. In 2017, FDA approved sarilumab for rheumatoid arthritis treatment (Khiali et al., 2021). It has reported that severe COVID-19 disease is characterized by elevated serum levels of C reactive protein (CRP) and cytokines, including, but not limited to, IFN-γ, IL-8, and IL-6 (Conti et al., 2020; Mo et al., 2020; Qin et al., 2020). Hence, this result provides a clue that anti-IL-6 agents have the possibility against SARS-CoV-2 infection. In a retrospective case report involving 15 COVID-19 patients, early intervention with sarilumab could have clinical improvement with decreased CRP level to patients with COVID-19 disease. More importantly, serum levels of CRP could be a potential biomarker for treatment response (Montesarchio et al., 2020). An open-label cohort study assessed the clinical outcome of sarilumab among 28 patients infected by SARS-CoV-2 compared with 28 contemporary patients receiving standard of care alone (Della-Torre et al., 2020). The results indicated that no significant difference was observed between sarilumab and standard of care. Of note, the clinical improvement suggested that sarilumab is relative to faster recovery in a subset of patients showing minor lung consolidation at baseline. In addition, there are several ongoing clinical trials to evaluate the effectiveness of sarilumab either plus standard of care (Caballero Bermejo et al., 2020) or combined with corticosteroids (ClinicalTrials.gov, NCT04357808) (Garcia-Vicuña et al., 2020) on COVID-19 disease. To date, the overall evaluation toward sarilumab on COVID-19 disease is much positive, which needs further tracking in the future.

### Stem cell-based therapy

To date, most studies regarding stem-based therapy to SARS-CoV-2 infection have focused on mesenchymal stem cells (MSCs) (Choudhery and Harris, 2020). MSC-based therapy has the ability to suppress the cytokine storm by secreting anti-inflammatory, anti-apoptosis, and antifibrosis cytokines. Also, MSCs contribute to antibacterial activity, as well as tissue repair and regeneration (Sadeghi et al., 2020). Table 8 shows clinical trials of MSCs on COVID-19 treatment. For patients suffering from COVID-19, MSCs would repair damaged alveolar epithelial cells and blood vessels, and also prevent pulmonary fibrosis (Chen et al., 2018; Leeman et al., 2019; Zanoni et al., 2019; Afra and Matin, 2020; Li Z. et al., 2020; Golchin et al., 2020). Seven COVID-19 patients who received intravenous transplantation of MSCs had significantly improved pulmonary function in 2 days after transplantation (Leng et al., 2020). Notably, the increased peripheral lymphocytes and IL-10 level, decreased C-reactive protein (CRP) and TNF-α level, and disappeared overactivated cytokine-secreting immune cells were observed within 14 days after MSC injection. Interestingly, Jayaramayya et al. reported that MSC-derived exosomes (MSC-Exo) may be an option to improve the clinical response to COVID-19 patients (Jayaramayya et al., 2020). A phase I clinical trial investigated the use of MSC-Exo inhalation to alleviate COVID-19-induced symptoms (clinicaltrials.gov, NCT04276987). Moreover, MSC-like derivatives have acceptable safety and efficacy for COVID-19 treatment in preclinical and clinical studies (Li Z. et al., 2020).

**TABLE 8:** Summary of clinical trials of mesenchymal stem cells (MSCs) on COVID-19 treatment.

Trial title	Sponsor	Trial phase	Primary intervention	Secondary intervention	Status	Identifier
Mesenchymal Stem Cell Infusion for COVID-19 Infection	Dr. Zaineb Akram	II	Mesenchymal stem cells	Placebo	Recruiting	NCT04444271
Treatment of COVID-19 Associated Pneumonia With Allogenic Pooled Olfactory Mucosa-Derived Mesenchymal Stem Cells	Institute of Biophysics and Cell Engineering of National Academy of Sciences of Belarus	I/II	Allogenic pooled olfactory mucosa-derived mesenchymal stem cells	Standard treatment	Enrolling by invitation	NCT04382547
Safety and Efficacy of Intravenous Wharton’s Jelly Derived Mesenchymal Stem Cells in Acute Respiratory Distress Syndrome Due to COVID 19	BioXcellerator	I/II	Wharton’s Jelly derived mesenchymal stem cells	Hydroxychloroquine, lopinavir/ritonavir or azithromycin and placebo	Recruiting	NCT04390152
Cord Blood-Derived Mesenchymal Stem Cells for the Treatment of COVID-19 Related Acute Respiratory Distress Syndrome	M.D. Anderson Cancer Center	I/II	Mesenchymal stem cells	Standard of care	Recruiting	NCT04565665
Mesenchymal Stem Cell for Acute Respiratory Distress Syndrome Due for COVID-19 (COVID-19)	Instituto Nacional de Ciencias Medicas y Nutricion Salvador Zubiran	II	Mesenchymal stem cells	N/A	Recruiting	NCT04416139
Bone Marrow-Derived Mesenchymal Stem Cell Treatment for Severe Patients With Coronavirus Disease 2019 (COVID-19)	Guangzhou Institute of Respiratory Disease	II	Bone marrow-derived mesenchymal stem cells	Placebo	Not yet recruiting	NCT04346368
Safety and Efficacy of Mesenchymal Stem Cells in the Management of Severe COVID-19 Pneumonia (CELMA)	Trustem	II	Umbilical cord derived mesenchymal stem cells	Placebo	Not yet recruiting	NCT04429763
NestaCell^®^ Mesenchymal Stem Cell to Treat Patients With Severe COVID-19 Pneumonia (HOPE)	Azidus Brasil	II	NestaCell^®^ mesenchymal stem cells	Placebo	Not yet recruiting	NCT04315987
Safety and Efficacy Study of Allogeneic Human Dental Pulp Mesenchymal Stem Cells to Treat Severe COVID-19 Patients	Renmin Hospital of Wuhan University	I/II	Allogeneic human dental pulp stem cells	Placebo	Recruiting	NCT04336254
Clinical Trial of Allogeneic Mesenchymal Cells From Umbilical Cord Tissue in Patients With COVID-19 (MESCEL-COVID19)	Hospital Infantil Universitario Niño Jesús, Madrid, Spain	II	Undifferentiated allogeneic mesenchymal cells derived from umbilical cord tissue	Standard of care	Recruiting	NCT04366271
Regenerative Medicine for COVID-19 and Flu-Elicited ARDS Using Longeveron Mesenchymal Stem Cells (LMSCs) (RECOVER) (RECOVER)	Longeveron LLC	I	Longeveron mesenchymal stem cells	Placebo	Recruiting	NCT04629105
Efficacy of Infusions of MSC From Wharton Jelly in the SARS-Cov-2 (COVID-19) Related Acute Respiratory Distress Syndrome (MSC-COVID19)	Central Hospital, Nancy, France	II	*Ex vivo* expanded Wharton’s Jelly mesenchymal stem cells	Placebo	Not yet recruiting	NCT04625738
Study to Evaluate the Efficacy and Safety of AstroStem-V in Treatment of COVID-19 Pneumonia	Nature Cell Co. Ltd	II	Allogenic adipose tissue-derived mesenchymal stem cells	N/A	Not yet recruiting	NCT04527224
Treatment With Human Umbilical Cord-Derived Mesenchymal Stem Cells for Severe Corona Virus Disease 2019 (COVID-19)	Beijing 302 Hospital	II	Human umbilical cord-mesenchymal stem cells	Placebo	Completed	NCT04288102
Treatment of Severe COVID-19 Patients Using Secretome of Hypoxia-Mesenchymal Stem Cells in Indonesia	Stem Cell and Cancer Research Indonesia	II	Secretome-mesenchymal stem cells + standard care	Standard treatment	Recruiting	NCT04753476
Mesenchymal Stem Cells in Patients Diagnosed With COVID-19	Hospital Reg. Lic. Adolfo Lopez Mateos	I	Mesenchymal stem cells	N/A	Recruiting	NCT04611256
Use of UC-MSCs for COVID-19 Patients	Camillo Ricordi	I/II	Umbilical cord mesenchymal stem cells + heparin along with best supportive care	Vehicle + heparin along with best supportive care	Completed	NCT04355728
Treatment of COVID-19 Patients Using Wharton’s Jelly-Mesenchymal Stem Cells	Stem Cells Arabia	I	Wharton’s Jelly derived mesenchymal stem cells	N/A	Recruiting	NCT04313322
Mesenchymal Stem Cell Treatment for Pneumonia Patients Infected With COVID-19	Beijing 302 Hospital	I	Mesenchymal stem cells + conventional treatment	Conventional treatment	Recruiting	NCT04252118
A Clinical Trial to Determine the Safety and Efficacy of Hope Biosciences Autologous Mesenchymal Stem Cell Therapy (HB-adMSCs) to Provide Protection Against COVID-19	Hope Biosciences	II	Autologous adipose-derived mesenchymal stem cells	N/A	Active, not recruiting	NCT04349631
Umbilical Cord Tissue (UC) Derived Mesenchymal Stem Cells (MSCs) Versus Placebo to Treat Acute Pulmonary Inflammation Due to COVID-19 (COVID-19)	Joshua M Hare	I	Umbilical cord tissue-derived mesenchymal stem cells	Placebo	Not yet recruiting	NCT04490486
Clinical Research of Human Mesenchymal Stem Cells in the Treatment of COVID-19 Pneumonia	Puren Hospital Affiliated to Wuhan University of Science and Technology	I/II	Umbilical cord tissue-derived mesenchymal stem cells	Placebo	Recruiting	NCT04339660
Study of Intravenous Administration of Allogeneic Adipose-Derived Mesenchymal Stem Cells for COVID-19-Induced Acute Respiratory Distress	Sorrento Therapeutics, Inc	II	Allogeneic adipose-derived mesenchymal stem cells	Placebo	Not yet recruiting	NCT04728698
Adipose Mesenchymal Cells for Abatement of SARS-CoV-2 Respiratory Compromise in COVID-19 Disease	Regeneris Medical	I	Autologous adipose-derived mesenchymal stem cells	N/A	Not yet recruiting	NCT04352803
A Randomized, Double-Blind, Placebo-Controlled Clinical Trial to Determine the Safety and Efficacy of Hope Biosciences Allogeneic Mesenchymal Stem Cell Therapy (HB-adMSCs) to Provide Protection Against COVID-19	Hope Biosciences	II	Allogeneic adipose-derived mesenchymal stem cells	Placebo	Active, not recruiting	NCT04348435
Mesenchymal Stem Cells for the Treatment of COVID-19	Thomas Advanced Medical LLC	I	PrimePro™ mesenchymal stem cells	Placebo	Completed	NCT04573270
Autologous Adipose-derived Stem Cells (AdMSCs) for COVID-19	Celltex Therapeutics Corporation	II	Autologous adipose-derived stem cells	Placebo	Not yet recruiting	NCT04428801
BAttLe Against COVID-19 Using MesenchYmal Stromal Cells	Instituto de Investigación Sanitaria de la Fundación Jiménez Díaz	II	Allogeneic and expanded adipose tissue-derived mesenchymal stromal cells	Regular treatment	Suspended (Lack of financial support.)	NCT04348461
Treatment of Coronavirus COVID-19 Pneumonia (Pathogen SARS-CoV-2) With Cryopreserved Allogeneic P_MMSCs and UC-MMSCs	Institute of Cell Therapy	I/II	Placenta-derived multipotent mesenchymal stromal cells + antibacterial (ceftriaxone, azithromycin), anticoagulants, hormones, oxygen therapy	Antibacterial (ceftriaxone, azithromycin), anticoagulants, hormones, oxygen therapy	Recruiting	NCT04461925
A Study of ADR-001 in Patients With Severe Pneumonia Caused by SARS-CoV-2 Infection (COVID-19)	Rohto Pharmaceutical Co., Ltd	II	Mesenchymal stem cells	Placebo	Not yet recruiting	NCT04888949
Umbilical Cord Lining Stem Cells (ULSC) in Patients With COVID-19 ARDS (ULSC)	Restem, LLC.	I/II	Umbilical cord lining stem cells	Placebo	Recruiting	NCT04494386
Therapeutic Study to Evaluate the Safety and Efficacy of DW-MSC in COVID-19 Patients (DW-MSC)	Ina-Respond	I	Allogeneic mesenchymal stem cells	Placebo	Completed	NCT04535856
Use of hUC-MSC Product (BX-U001) for the Treatment of COVID-19 With ARDS	Baylx Inc	I/II	Human umbilical cord mesenchymal stem cells + supportive care	Placebo control + supportive care	Not yet recruiting	NCT04452097
Efficacy and Safety Study of Allogeneic HB-adMSCs for the Treatment of COVID-19	Hope Biosciences	II	Allogeneic adipose-derived mesenchymal stem cells	Placebo	Active, not recruiting	NCT04362189
Clinical Use of Stem Cells for the Treatment of COVID-19	SBÜ Dr. Sadi Konuk Eğitim ve Araştırma Hastanesi	I	Mesenchymal stem cells	Placebo	Recruiting	NCT04392778
Mesenchymal Stromal Cells for the Treatment of SARS-CoV-2 Induced Acute Respiratory Failure (COVID-19 Disease)	Baylor College of Medicine	I/II	Mesenchymal stem cells	Supportive care	Recruiting	NCT04345601
Efficacy and Safety Evaluation of Mesenchymal Stem Cells for the Treatment of Patients with Respiratory Distress Due to COVID-19 (COVIDMES)	Banc de Sang i Teixits	I/II	Wharton’s Jelly derived mesenchymal stem cells	Placebo	Recruiting	NCT04390139
Mesenchymal Stem Cells (MSCs) in Inflammation-Resolution Programs of Coronavirus Disease 2019 (COVID-19) Induced Acute Respiratory Distress Syndrome (ARDS)	University Hospital Tuebingen	II	Mesenchymal stem cells	N/A	Not yet recruiting	NCT04377334
Study of the Safety of Therapeutic Tx With Immunomodulatory MSC in Adults With COVID-19 Infection Requiring Mechanical Ventilation	ImmunityBio, Inc	I	Immunomodulatory mesenchymal stem cells	Placebo	Recruiting	NCT04397796
The Use of Exosomes for the Treatment of Acute Respiratory Distress Syndrome or Novel Coronavirus Pneumonia Caused by COVID-19 (ARDOXSO)	AVEM HealthCare	I/II	Mesenchymal stem cell—exosomes	N/A	Not yet recruiting	NCT04798716
A phase II Study in Patients With Moderate to Severe ARDS Due to COVID-19	Stemedica Cell Technologies, Inc	II	Allogeneic mesenchymal stem cells	Placebo	Recruiting	NCT04780685
Repair of Acute Respiratory Distress Syndrome by Stromal Cell Administration (REALIST) (COVID-19) (REALIST)	Belfast Health and Social Care Trust	I/II	Human umbilical cord-derived CD362-enriched mesenchymal stem cells	Placebo	Recruiting	NCT03042143
Safety and Feasibility of Allogenic MSC in the Treatment of COVID-19 (COVID19)	Hospital de Clinicas de Porto Alegre	I	Mesenchymal stem cells	N/A	Not yet recruiting	NCT04467047
Efficacy of Intravenous Infusions of Stem Cells in the Treatment of COVID-19 Patients	Jinnah Hospital	II	Umbilical cord-derived mesenchymal stem cells + standard care	Standard care	Recruiting	NCT04437823
Treatment of Severe COVID-19 Pneumonia With Allogeneic Mesenchymal Stromal Cells (COVID_MSV) (COVID_MSV)	Red de Terapia Celular	II	Mesenchymal stromal cells	Placebo	Recruiting	NCT04361942
Multiple Dosing of Mesenchymal Stromal Cells in Patients With ARDS (COVID-19)	Masonic Cancer Center, University of Minnesota	II	Mesenchymal stromal cells	Placebo	Active, not recruiting	NCT04466098
Cellular Immuno-Therapy for COVID-19 Acute Respiratory Distress Syndrome (CIRCA-19)	Ottawa Hospital Research Institute	I/II	Mesenchymal Stromal Cells	N/A	Completed	NCT04400032

(All information in the table are collected from https://clinicaltrials.gov).

However, some limitations remain to be considered (Sadeghi et al., 2020). First, some patients with, including, but not limited to, a history of malignant tumor, coinfections of other respiratory viruses, and pregnant woman are not eligible to evolve in clinical trials. Most clinical trials worldwide remain in phase I and II, and comprehensive results are not clear. Furthermore, it is difficult to evaluate the effectiveness of MSC therapy alone when coadministration with other conventional drugs, such as remdesivir or dexamethasone, in many cases. Importantly, the standard therapeutic protocol, such as administration route, dosage, and transplantation frequency, needs to be determined. Nevertheless, the MSC profile on the immune system provides researchers evidence that it may be a good candidate as a combination therapy of infectious diseases such as COVID-19. Overall, MSC-based therapy appears to be a potential and promising therapeutic method to overcome SARS-CoV-2 infection.

### Convalescence plasma transfusion

Convalescent plasma treatment provides immediate immunity by passive polyclonal antibody administration (Mair-Jenkins et al., 2015). The efficacy of convalescent plasma transfusion may result from viremia suppression (Chen L. et al., 2020). It has reported that convalescent plasma treatment can be used to improve the survival rate on patients with severe acute respiratory syndromes of viral etiology (Mair-Jenkins et al., 2015). Several studies indicated that SARS patients who were treated with convalescent plasma had a shorter hospital stay and lower mortality than those who were not treated with convalescent plasma (Soo et al., 2004; Cheng et al., 2005; Lai, 2005). Table 9 shows the clinical trials of convalescent plasma transfusion on COVID-19 treatment. Based on the findings from recent studies, initiating treatment no later than 5 days may be the most appropriate (Woelfel et al., 2020; Zhao et al., 2020b). Tiberghien et al. (2020) recommend that convalescent plasma administration at the early phases of the disease in patients at high risk of deleterious evolution may reduce the frequency of patient deterioration and, thereby, COVID-19 mortality. Also, close monitoring is necessary to detect any unintended side effects. However, a randomized trial (clinicaltrials.gov, NCT04383535) evolved in 228 COVID-19 patients to evaluate the clinical status after convalescent plasma intervention was added to standard treatment (Simonovich et al., 2021). Unfortunately, no significant differences were found in clinical outcomes or overall mortality between patients infused with convalescent plasma added to standard treatment and those who received standard treatment alone within 30 days. Similarly, an open-label, multicenter, randomized clinical trial (www.chictr.org.cn, ChiCTR2000029757) was performed in seven medical centers with 103 COVID-19 patients (Li L. et al., 2020). The results showed that convalescent plasma therapy in addition to standard treatment, compared with standard treatment alone, did not result in a significant improvement in time to clinical improvement within 28 days. Of note, it is known that other treatments, including antiviral drugs, steroids, and intravenous immunoglobulin, have the possibility to affect the relationship between convalescent plasma and antibody level (Luke et al., 2006). Thus, it is controversial whether it is worthwhile to examine the safety and efficacy of convalescent plasma intervention against SARS-CoV-2 infection in further randomized clinical trials.

**TABLE 9:** Summary of clinical trials of convalescence plasma on COVID-19 treatment.

Trial title	Sponsor	Trial phase	Primary intervention	Secondary intervention	Status	Identifier
Safety in Convalescent Plasma Transfusion to COVID-19	Hospital San Jose Tec de Monterrey	I	Convalescent plasma	N/A	Terminated (Other clinical trials probed that the use of convalescent plasma for patients with COVID-19 is safe.)	NCT04333355
Convalescent Plasma (PC) and Human Intravenous Anti-COVID-19 Immunoglobulin (IV Anti COVID-19 IgG) in Patients Hospitalized for COVID-19	Lifefactors Zona Franca, SAS	II/III	Convalescent plasma	Anti-COVID-19 human immunoglobulin; standard therapy (remdesivir, chloroquine, hydroxychloroquine, azithromycin)	Not yet recruiting	NCT04395170
Convalescent Plasma of COVID-19 to Treat SARS-COV-2 a Randomized Doble Blind 2 Center Trial (CPC-SARS)	Grupo Mexicano para el Estudio de la Medicina Intensiva	II	Convalescent plasma + conventional therapy (azithromycin and hydroxychloroquine)	Conventional therapy (azithromycin and hydroxychloroquine) and 20% albumin	Completed	NCT04405310
Convalescent Plasma Therapy in Severe COVID-19 Infection	Bangabandhu Sheikh Mujib Medical University, Dhaka, Bangladesh	II	Standard supportive treatment	Standard treatment + convalescent plasma	Recruiting	NCT04403477
Convalescent Plasma Therapy in Patients With COVID-19	Biofarma	I	Convalescent plasma	N/A	Completed	NCT04407208
Convalescent Plasma for Ill Patients by COVID-19 (COPLASCOV19)	Instituto de Seguridad y Servicios Sociales de los Trabajadores del Estado	I/II	Convalescent plasma	N/A	Recruiting	NCT04356482
COVID19-Convalescent Plasma for Treating Patients With Active Symptomatic COVID 19 Infection (FALP-COVID) (FALP-COVID)	Fundacion Arturo Lopez Perez	II/III	Convalescent plasma	N/A	Recruiting	NCT04384588

(All information in the table are collected from https://clinicaltrials.gov).

### Vaccines

An efficacious vaccine is critical to prevent morbidity and mortality caused by COVID-19. There are four categories of COVID-19 vaccines under clinical evaluation, including whole-pathogen vaccines (inactivated vaccines), subunit vaccines, and nucleic acid (DNA and mRNA) vaccines. However, defining and assessing an efficacious vaccine is complex. In the case of SARS-CoV-2 infection, an efficacious vaccine could reduce the likelihood of an infection in an individual, severity of a disease in an individual, or the degree of transmission within a population (Hodgson et al., 2021). The comprehensive understanding of SARS-Cov-2 is unclear and evolving, thereby the outcomes for a COVID-19 vaccine are critically appraised with scientific rigor to understand their generalizability and clinical significance.

Currently, three vaccines are authorized in the United States: Pfizer-BioNTech (Name: BNT162b2), Moderna (Name: mRNA-1273), and Johnson and Johnson/Janssen (Name: JNJ-78436735). Tables 10–12 summarize the clinical trials of these vaccines for the treatment of COVID-19. Of note, people under 12 years old are not eligible to receive vaccine produced by Pfizer-BioNTech, and people under 18 years old are not eligible to receive vaccines produced by Moderna and Johnson and Johnson/Janssen. Kamidani et al. (2021) indicated that children are supposed to have the opportunity to be included in clinical trials in parallel to ongoing adult phase III clinical trials. It is because the development of a pediatric COVID-19 vaccine could prevent disease and alleviate downstream effects including social isolation and interruption in education, thereby enabling children to re-engage in their world. Considering the SARS-CoV-2 variants, evidence from Polack et al. (2020) proved that BNT162b2 is 95% effective against SARS-CoV-2 infection. A 6 months of follow-up evaluation from Thomas et al. (2021) indicated that BNT162b2 has a favorable safety profile and effectively prevents COVID-19 for up to 6 months including the beta variant even though there is a gradual decline in effectiveness. Bernal et al. (Lopez Bernal et al., 2021) reported that the efficacy of the one-shot BNT162b2 vaccine is 30.7% among individuals with the delta variant, while the efficacy is 48.7% among individuals with the alpha variant. The efficacy of two shots of BNT162b2 vaccine is 88.0% among individuals with the delta variant, while the efficacy is 93.7% among individuals with the alpha variant. In other words, as CDC recommendation, vaccination against COVID-19 is the best way to stop the spread of these predominate COVID-19 strains.

**TABLE 10:** Summary of clinical trials of BNT162b2 vaccine (produced by Pfizer-BioNTech) on COVID-19 treatment.

Trial title	Sponsor	Trial phase	Primary intervention	Secondary intervention	Status	Identifier
Safety and Immunogenicity Study of 20vPnC When Coadministered With a Booster Dose of BNT162b2	Pfizer	III	An injection of pneumococcal vaccine (20vPnC) and of COVID-19 vaccine (BNT162b2) at the same visit	An injection of pneumococcal vaccine (20vPnC) alone; an injection of COVID-19 vaccine (BNT162b2) alone	Active, not recruiting	NCT04887948
Study to Evaluate the Safety, Tolerability, and Immunogenicity of Multiple Formulations of BNT162b2 Against COVID-19 in Healthy Adults	BioNTech SE	III	BNT162b2	N/A	Active, not recruiting	NCT04816669
A Trial Investigating the Safety and Effects of One or Two Additional Doses of Comirnaty or One Dose of BNT162b2s01 in BNT162-01 or BNT162-04 Trial Subjects	BioNTech SE	II	BNT162b2	N/A	Recruiting	NCT04949490
Study to Evaluate the Safety and Efficacy of a Booster Dose of BNT162b2 Against COVID-19 in Participants ≥16 Years of Age	BioNTech SE	III	BNT162b2	Placebo	Recruiting	NCT04955626
Study to Evaluate Safety, Tolerability and Immunogenicity of BNT162b2 in Immunocompromised Participants ≥2 Years	BioNTech SE	II	BNT162b2	N/A	Not yet recruiting	NCT04895982
Study to Evaluate the Safety, Tolerability, and Immunogenicity of SARS CoV-2 RNA Vaccine Candidate (BNT162b2) Against COVID-19 in Healthy Pregnant Women 18 Years of Age and Older	BioNTech SE	III	BNT162b2	Placebo	Recruiting	NCT04754594
A phase 3 Study to Evaluate the Safety, Tolerability, and Immunogenicity of Multiple Production Lots and Dose Levels of BNT162b2 RNA-Based COVID-19 Vaccines Against COVID-19 in Healthy Participants	BioNTech SE	III	BNT162b2	N/A	Completed	NCT04713553
Safety and Immunogenicity of SARS-CoV-2 mRNA Vaccine (BNT162b2) in Chinese Healthy Population	BioNTech SE	II	BNT162b2	Placebo	Active, not recruiting	NCT04649021
Third Dose of mRNA Vaccination to Boost COVID-19 Immunity	The University of Hong Kong	IV	BNT162b2	N/A	Recruiting	NCT05057182
Impact of the Immune System on Response to Anti-Coronavirus Disease 19 (COVID-19) Vaccine in Allogeneic Stem Cell Recipients (Covid Vaccin Allo)	University of Liege	III	BNT162b2	N/A	Recruiting	NCT04951323
Study to Evaluate the Safety, Tolerability, and Immunogenicity of an RNA Vaccine Candidate Against COVID-19 in Healthy Japanese Adults	BioNTech SE	I/II	BNT162b2	Placebo	Active, not recruiting	NCT04588480
Booster Dose of COVID-19 Vaccine for Kidney Transplant Recipients Without Adequate Humoral Response (WHO)	Dafna Yahav	IV	BNT162b2	N/A	Not yet Recruiting	NCT04961229
Study to Describe the Safety, Tolerability, Immunogenicity, and Efficacy of RNA Vaccine Candidates Against COVID-19 in Healthy Individuals	BioNTech SE	II/III	BNT162b2	Placebo	Recruiting	NCT04368728
Randomized Trial of COVID-19 Booster Vaccinations (Cobovax Study)	The University of Hong Kong	IV	BNT162b2	CoronaVac	Not yet recruiting	NCT05057169
Study to Evaluate the Safety, Tolerability, and Immunogenicity of an RNA Vaccine Candidate Against COVID-19 in Healthy Children <12 Years of Age	BioNTech SE	II/III	BNT162b2	N/A	Recruiting	NCT04816643
Safety and Immunogenicity of a SARS CoV 2 Multivalent RNA Vaccine in Healthy Participants	BioNTech SE	II	BNT162b2	N/A	Recruiting	NCT05004181
Third Dose Vaccination with AstraZeneca or Pfizer COVID-19 Vaccine Among Adults Received Sinovac COVID-19 Vaccine	Mahidol University	II	BNT162b2	ChAdOx1 AZD1222	Not yet recruiting	NCT05049226
Vaccination for Recovered Inpatients With COVID-19 (VATICO)	International Network for Strategic Initiatives in Global HIV Trials (INSIGHT)	IV	BNT162b2	mRNA-1273	Recruiting	NCT04969250
Mix and Match of the Second COVID-19 Vaccine Dose for Safety and Immunogenicity (MOSAIC)	Canadian Immunization Research Network	II	BNT162b2	mRNA-1273; ChAdOx1-S	Recruiting	NCT04894435
COVID-19 Booster Vaccine in Autoimmune Disease Non-Responders	National Institute of Allergy and Infectious Diseases (NIAID)	II	BNT162b2	mRNA-1273; Ad26.COV2.S	Recruiting	NCT05000216

(All information in the table are collected from https://clinicaltrials.gov).

**TABLE 11:** Summary of clinical trials of mRNA-1273 vaccine (produced by Moderna) on COVID-19 treatment.

Trial title	Sponsor	Trial phase	Primary intervention	Secondary intervention	Status	Identifier
A Study to Evaluate the Safety, Reactogenicity, and Effectiveness of mRNA-1273 Vaccine in Adolescents 12 to <18 Years Old to Prevent COVID-19 (TeenCove)	ModernaTX, Inc	II/III	mRNA-1273	Placebo	Active, not recruiting	NCT04649151
A Study to Evaluate Safety and Effectiveness of mRNA-1273 COVID-19 Vaccine in Healthy Children Between 6 Months of Age and Less Than 12 Years of Age	ModernaTX, Inc	II/III	mRNA-1273	Placebo	Recruiting	NCT04796896
A Study to Evaluate Safety and Immunogenicity of mRNA-1273 Vaccine to Prevent COVID-19 in Adult Organ Transplant Recipients and in Healthy Adult Participants	ModernaTX, Inc	III	mRNA-1273	N/A	Recruiting	NCT04860297
A Study to Evaluate Efficacy, Safety, and Immunogenicity of mRNA-1273 Vaccine in Adults Aged 18 Years and Older to Prevent COVID-19	ModernaTX, Inc	III	mRNA-1273	Placebo	Active, not recruiting	NCT04470427
Safety and Immunogenicity Study of 2019-nCoV Vaccine (mRNA-1273) for Prophylaxis of SARS-CoV-2 Infection (COVID-19)	National Institute of Allergy and Infectious Diseases (NIAID)	I	mRNA-1273	N/A	Active, not recruiting	NCT04283461
Study About the Response to the Administration of a Third Dose of mRNA-1273 Vaccine (COVID-19 Vaccine Moderna) in Renal Transplants With Immunological Failure Initial to Vaccination (VAX-TRES)	Maria Joyera Rodríguez	II	mRNA-1273	N/A	Not yet recruiting	NCT04930770
Third Dose of COVID-19 Vaccine in LTCF Residents	Mark Loeb	IV	mRNA-1273	Prevnar13	Not yet recruiting	NCT04978038
Dose-Confirmation Study to Evaluate the Safety, Reactogenicity, and Immunogenicity of mRNA-1273 COVID-19 Vaccine in Adults Aged 18 Years and Older	ModernaTX, Inc	II	mRNA-1273	Placebo	Active, not recruiting	NCT04405076
A Study to Evaluate Safety, Reactogenicity, and Immunogenicity of mRNA-1283 and mRNA-1273 Vaccines in Healthy Adults Between 18 and 55 Years of Age to Prevent COVID-19	ModernaTX, Inc	I	mRNA-1273	Placebo	Active, not recruiting	NCT04813796
Immunogenecity and Safety of VaccinemRNA-1273 in Elderly Volunteers (Over 65 years) Compared to Younger Ones (18–45 years) (CoviCompareM)	Assistance Publique - Hôpitaux de Paris	II	mRNA-1273	N/A	Not yet recruiting	NCT04748471
A Study to Evaluate the Immunogenicity and Safety of mRNA-1273.211 Vaccine for COVID-19 Variants	ModernaTX, Inc	II/III	mRNA-1273	mRNA-1273.211; mRNA-1273.617.2	Active, not recruiting	NCT04927065
Safety and Immunogenicity Study of a SARS-CoV-2 (COVID-19) Variant Vaccine (mRNA-1273.351) in Naïve and Previously Vaccinated Adults	National Institute of Allergy and Infectious Diseases (NIAID)	I	mRNA-1273	mRNA-1273.351	Active, not recruiting	NCT04785144
RECOVAC Booster Vaccination Study	University Medical Center Groningen	IV	mRNA-1273	Ad26.COV2.S vaccine	Not yet recruiting	NCT05030974
Third Dose of Moderna COVID-19 Vaccine in Transplant Recipients	University Health Network, Toronto	IV	mRNA-1273	Placebo	Active, not recruiting	NCT04885907
Delayed Heterologous SARS-CoV-2 Vaccine Dosing (Boost) After Receipt of EUA Vaccines	National Institute of Allergy and Infectious Diseases (NIAID)	I/II	mRNA-1273	mRNA-1273.211; Ad26.COV2.S; BNT162b2	Recruiting	NCT04889209
SARS-CoV-2 Immune Responses After COVID-19 Therapy and Subsequent Vaccine	National Institute of Allergy and Infectious Diseases (NIAID)	IV	mRNA-1273	N/A	Recruiting	NCT04952402

(All information in the table are collected from https://clinicaltrials.gov).

**TABLE 12:** Summary of clinical trials of JNJ-78436735 vaccine (produced by Johnson and Johnson/Janssen) on COVID-19 treatment.

Trial title	Sponsor	Trial phase	Primary intervention	Secondary intervention	Status	Identifier
COVID-19 3rd Dose Vaccine in Transplant Patients	Giselle Guerra	III	BNT162b2	JNJ-78436735	Not yet recruiting	NCT05047640

(All information in the table are collected from https://clinicaltrials.gov).

Most recently, a COVID-19 vaccine booster emerged to help individuals build enough protection after vaccination. According to the information from Centers for Disease Control and Prevention (CDC, https://www.cdc.gov), individuals who have received their second dose of an mRNA COVID-19 vaccine (produced by either Pfizer-BioNTech or Moderna) for 8 months are eligible to get a booster shot. Currently, for individuals who got Johnson and Johnson/Janssen vaccine, there is not enough data to support getting an mRNA vaccine dose.

## Traditional Chinese medicine

Xuebijing injection (XBJ) consists of *Carthamus tinctorius* L., *Paeonia lactiflora* Pall., *Ligusticum striatum* DC., *Salvia miltiorrhiza* Bunge, and *Angelica sinensis* (Oliv.) Diels (Shi et al., 2017). XBJ constructs a “drug-ingredient-target-pathway” effector network to exert its therapeutic effects on COVID-19 prevention and treatment (Zheng et al., 2020). Guo et al. (2020) conducted a retrospective case-control study to determine the efficacy of XBJ on SARS-CoV-2 infection with 42 patients who received routine treatment combined with XBJ (observation group) and 16 patients who received routine treatment alone (control group). The results showed that patients in the observation group had a significant reduction in body temperature, improvement in CT imaging results, and shorter time in a negative nucleic acid test recovery relative to those in the control group. Also, improvement in IL-6 levels was found in the observation group compared with those in the control group, while TNF-α and IL-10 levels did not show significant differences between the two groups. In addition, 284 COVID-19 patients were enrolled in a multicenter, prospective, randomized controlled trial to assess the effectiveness of Lianhuaqingwen (LH) capsule (Hu et al., 2021). Compared with patients in the control group (received usual treatment alone), patients with usual treatment in combination with LH capsule treatment had higher recovery rate, shorter median time to symptom recovery, and higher rate of improvements in chest CT manifestations and clinical cure. Hence, both XBJ and LH capsules could be considered to ameliorate clinical symptoms of COVID-19. Moreover, Ni et al. reported that using Western medicine combined with Chinese traditional patent medicine Shuanghuanglian oral liquid (SHL) has expected therapeutic outcomes to COVID-19 patients, and thereby warrants further clinical trials (Ni et al., 2020b).

## Concluding remarks

For antimicrobial drugs, the acquired drug resistance should be considered and explored. The use of CQ and HCQ is controversial due to their toxicity and side effects. Moreover, lopinavir/ritonavir, umifenovir, and azithromycin appear to be promising therapeutic drugs even though some studies do not show ideal and unfavorable clinical outcomes on COVID-19 patients. The IFNs are usually used in addition to other antiviral drugs. Also, the application of IFN-λ have more advantages than other types of IFNs in COVID-19 treatment.

TCZ, an antibody, has the ability to improve clinical responses on COVID-19 patients by suppressing inflammatory storm and, thereby, reduces mortality cases. Mepolizumab, as an antibody medication for asthma, may increase the risk of severe COVID-19 and induce a more severe course of COVID-19, particularly for COVID-19 patients with severe asthma receiving mepolizumab therapy. Sarilumab, as an FDA-approved antibody medication for rheumatoid arthritis treatment, shows clinical improvement with decreased CRP level to patients with COVID-19 disease. Furthermore, stem cell-based therapy, especially MSCs, could improve clinical symptoms and repair tissue caused by SARS-CoV-2 infection. Of note, the standard protocol of MSCs therapy needs to be determined. Additionally, COVID-19 patients who received convalescent plasma transfusion in addition to standard treatment shows no clinical differences compared with those who received standard treatment alone. Therefore, it is controversial whether it is worthwhile to assess the safety and efficacy of convalescent plasma intervention against SARS-CoV-2 infection in further randomized clinical trials.

In addition, TCMs play a critical role in ameliorating and alleviating clinical symptoms on COVID-19 patients. Also, it is known that TCMs in combination with Western medicine is a potential therapeutic strategy against SARS-CoV-2 infection.

To date, remdesivir is FDA approved specifically for the treatment of COVID-19. Also, several vaccines are authorized and recommended in the United States and other countries. Most treatment regimens against the COVID-19 pandemic are controversial and remain under preclinical and clinical trials. Overall, more comprehensive information regarding each treatment regimen is uncertain and needs to be further explored.
